# Characterization of *In Vivo* Dlg1 Deletion on T Cell Development and Function

**DOI:** 10.1371/journal.pone.0045276

**Published:** 2012-09-18

**Authors:** Lisa A. Humphries, Meredith H. Shaffer, Faruk Sacirbegovic, Tamar Tomassian, Kerrie-Ann McMahon, Patrick O. Humbert, Oscar Silva, June L. Round, Kogo Takamiya, Richard L. Huganir, Janis K. Burkhardt, Sarah M. Russell, M. Carrie Miceli

**Affiliations:** 1 Department of Microbiology, Immunology, and Molecular Genetics, University of California Los Angeles, Los Angeles, California, United States of America; 2 Department of Pathology and Laboratory Medicine, Children’s Hospital of Philadelphia and Perelman School of Medicine at the University of Pennsylvania, Philadelphia, Pennsylvania, United States of America; 3 Immune Signalling Laboratory, Peter MacCallum Cancer Centre, East Melbourne, Victoria, Australia; 4 Department of Pathology, University of Melbourne, Melbourne, Victoria, Australia; 5 Molecular Biology Institute, University of California Los Angeles, Los Angeles, California, United States of America; 6 Cell Cycle and Cancer Genetics Laboratory, Peter MacCallum Cancer Centre, East Melbourne, Victoria, Australia; 7 Sir Peter MacCallum Department of Oncology, University of Melbourne, Melbourne, Victoria, Australia; 8 Center for Micro-Photonics, Faculty of Engineering and Industrial Sciences, Swinburne University of Technology, Melbourne, Victoria, Australia; 9 Department of Neuroscience, Faculty of Medicine, University of Miyazaki, Miyazaki, Japan; 10 Department of Neuroscience, Howard Hughes Medical Institute and Brain Science Institute, The Johns Hopkins University School of Medicine, Baltimore, Maryland, United States of America; New York University, United States of America

## Abstract

**Background:**

The polarized reorganization of the T cell membrane and intracellular signaling molecules in response to T cell receptor (TCR) engagement has been implicated in the modulation of T cell development and effector responses. In siRNA-based studies Dlg1, a MAGUK scaffold protein and member of the Scribble polarity complex, has been shown to play a role in T cell polarity and TCR signal specificity, however the role of Dlg1 in T cell development and function *in vivo* remains unclear.

**Methodology/Principal Findings:**

Here we present the combined data from three independently-derived *dlg1*-knockout mouse models; two germline deficient knockouts and one conditional knockout. While defects were not observed in T cell development, TCR-induced early phospho-signaling, actin-mediated events, or proliferation in any of the models, the acute knockdown of Dlg1 in Jurkat T cells diminished accumulation of actin at the IS. Further, while Th1-type cytokine production appeared unaffected in T cells derived from mice with a *dlg1*germline-deficiency, altered production of TCR-dependent Th1 and Th2-type cytokines was observed in T cells derived from mice with a conditional loss of *dlg1* expression and T cells with acute Dlg1 suppression, suggesting a differential requirement for Dlg1 activity in signaling events leading to Th1 versus Th2 cytokine induction. The observed inconsistencies between these and other knockout models and siRNA strategies suggest that 1) compensatory upregulation of alternate gene(s) may be masking a role for *dlg1* in controlling TCR-mediated events in *dlg1* deficient mice and 2) the developmental stage during which *dlg1* ablation begins may control the degree to which compensatory events occur.

**Conclusions/Significance:**

These findings provide a potential explanation for the discrepancies observed in various studies using different *dlg1*-deficient T cell models and underscore the importance of acute *dlg1* ablation to avoid the upregulation of compensatory mechanisms for future functional studies of the Dlg1 protein.

## Introduction

T cell development and effector function is dependent on the ability of T cells to dynamically polarize and selectively rearrange membrane and actin cytoskeletal components to mediate cell:cell interactions, trafficking to lymphoid compartments and sites of infection, and responses to antigen recognition [1]–[5]. Effective recognition of antigen by the T cell receptor (TCR) is an essential event for T cell activation in the context of an antigen-presenting cell (APC) and a critical control point for the development and regulation of adaptive immunity. Following TCR-mediated recognition of its cognate peptide, a T cell undergoes rapid and dynamic cytoskeletal and membrane reorganization which facilitates the polarized recruitment and segregation of cellular components into specialized macromolecular assemblies: the immunological synapse (IS), located at the APC:T-cell interface, and the distal pole complex (DPC), formed opposite the [6], [7].

The IS serves as a multi-tasking platform for coupling TCR proximal non-receptor tyrosine kinases, Lck and ZAP-70, to downstream transducer pathways. It has been suggested that recruitment of particular transducers at the synapse specifies distinct transcriptional activation profiles to selectively direct T cell functional outcome [8], [9]. Furthermore, the IS directs reorientation of the microtubule-organizing center (MTOC) and the polarized trafficking of cytokines, cytolytic granules and fate determinants, to promote directional secretion, targeted cell killing and asymmetric cell division [1], [10]–[12]. Distal to the APC:T cell contact site, DPC assembly may promote T cell activation by sequestering negative regulators away from the IS, or may orchestrate asymmetric cell division [7], [13], [14].

Formation of both the IS and DPC have been shown to correlate with the asymmetrical distribution of polarity proteins [3] providing a mechanism for diversifying signals for effector activity as well as to specify fate. The composition of the IS and DPC can vary in different T cell subsets and throughout activation [15]–[17] and these complexes have been shown to regulate T cell effector responses and fate decisions through the selective recruitment and juxtaposition of surface receptors, intracellular signal transducers, negative regulators, and cytoskeletal/membrane components into discrete functional domains [4], [18], [19]. It has been proposed that the polarization of activated T cells and resulting distribution of key signaling molecules and cellular machinery within the IS and DPC may also guide memory and effector fate decisions [20]. Indeed, asymmetric proteasome segregation to the DPC results in unequal partitioning of the transcription factor T-bet during T cell division and the generation of functionally distinct daughter populations [11]. Understanding the mechanisms that control and regulate T lymphocyte polarity may thus lend insight into methods by which T cell effector responses and memory development can be manipulated in a targeted fashion.

Lymphocyte cell polarity is orchestrated by evolutionarily conserved protein networks including the Scribble, PAR (partitioning-defective), and Crumbs complexes [3]. These ancestral polarity complexes are well characterized as master regulators of epithelial cell apico-basal polarity. The Scribble complex, composed of the Scribble, Lethal giant larvae (Lgl) and Discs large (Dlg) proteins, regulates epithelial polarity by recruiting surface receptors and signaling molecules through interaction with the cytoskeleton and other structural elements [21]. Similarly, Scribble complex proteins, including Dlg1, localize at the IS and DPC in an orchestrated manner and both Scribble and Dlg1 have been shown to regulate IS and DPC functions, including T cell morphology and migration [3], [7], [22], [23].

Dlg1, (hDlg/Dlgh1, Synapse-associated protein 97/SAP97), is a founding member of the membrane associated guanylate kinase (MAGUK) family, a multi-domain scaffolding protein which associates with signaling and cytoskeletal effector molecules important for T cell signal transduction and polarity. Structurally, Dlg1 is composed of an N-terminal L27β oligomerization domain, a proline-rich domain (PRD), three PDZ (PSD-95, Dlg, and ZO-1) domains, an SH3 (Src Homology 3) domain and a catalytically-inactive GUK (GUanylate Kinase) domain. During antigen recognition, these modular domains allow Dlg1 to co-localize with synaptic actin, translocate into sphingolipid-rich microdomains within the IS and associate with Lck, ZAP-70, Vav, WASp, Ezrin and p38 [3], [23]–[25], although the mode of regulation and functional significance of several of these interactions remain unclear. The association of Dlg1 with cytoskeletal regulators WASp and Ezrin is hypothesized to facilitate T cell polarity by coupling TCR engagement to actin polymerization, the clustering of synaptic TCRs, and MTOC polarization. Knockdown of Dlg1 expression attenuates TCR triggered F-actin polymerization and the polarized recruitment of lipid rafts, TCR and the MTOC to the IS [22], processes known to utilize WASp and Ezrin [2], [26].

T cell functional outputs are regulated through integrating signal transduction pathways and cytoskeletal reorganization events initiated and maintained by TCR and co-receptor engagement to allow for proper magnitude, duration and type of effector response. TCR stimulation triggers the juxtaposition of Lck, ZAP-70 and p38 by Dlg1. This unique arrangement facilitates direct phosphorylation of p38 by ZAP-70, promoting p38 autophosphorylation [24], [27] through a process referred to as alternative p38 activation. Dlg1-mediated alternative p38 activation selectively activates the nuclear factor of activated T cells (NFAT), but not nuclear factor κB (NFκB), due to the direct or indirect phosphorylation of the NFAT transactivation domain [24], [26], [28], affecting NFAT substrate specificity and directing its activity to a discrete set of downstream targets [29]. In line with these data, knockdown of Dlg1 expression in primary antigen-experienced CD8+ T cells disrupts TCR-induced cytokine production and contact-dependent cytolysis [22]. However, in one report, the overexpression of Dlg1in the presence of Vav in Jurkat T cells was found to impair NFAT activity [23], suggesting that Dlg1 activity may differ in particular T cell contexts or function as a dominant negative when expressed at high levels. While the mechanisms by which Dlg1 regulates these effector responses have not been completely elucidated, Dlg1 binding partners p38, WASp and Ezrin have all been implicated in processes that affect cytokine production and/or cytotoxicity [26], [30]–[34].

While previous studies have implicated Dlg1 in regulating T cell signaling, polarity and effector responses, the majority of reports on Dlg1 in T cells have utilized siRNA-mediated knockdown strategies [22]–[24], [26], [28]. However, knockdown technology is limited, confining studies to activated T cells over a transient period of time and precluding the evaluation of T cell development. Moreover, since knockdown is often incomplete, it does not allow for analysis in a true null background. Thus, to build on and extend the current understanding of Dlg1 in T cell development, signaling, and actin-cytoskeletal events, three distinct *dlg1* deficient mouse strains were independently characterized by three groups. Here, we present the combined cellular and biochemical analyses of one conditional and two germline *dlg1* knockout mouse models from these groups. In total these data show that the ablation of *dlg1* expression in T cells in these independently-derived mouse models is largely unremarkable, with no, or minor, observable defects in T cell development, morphology, migration, signaling and/or proliferation. Nonetheless, the different knockout models do lead to subtle differences in T cell functionality, with the most significant effects observed in the model in which *dlg1* was knocked out conditionally during T cell development. These findings are consistent with suggestions that compensatory mechanisms may come into play at different stages of T cell development and mask Dlg1 function in T cell populations with long term Dlg1 ablation.

## Results

### Dlg1 is a Discs Large-family Member Expressed in T Cells

To examine which *dlg* genes are expressed in mouse T cells, specific primer sets were used to amplify a small region of four of the seven *dlg* transcripts, *dlg1, dlg2 (PSD-93), dlg3 (NE-dlg),* and *dlg4* (PSD-95) from murine mRNA. The expression of the distantly related Dlg family members (*dlg5*–*dlg7*) was not determined. While mRNA from *dlg1, 2, 3 and 4* was detected in murine brain, only *dlg1* and *dlg4* were detected in murine T cells (Figure S1A). Previous studies have confirmed the presence of Dlg1 [3], [22] and Dlg4 [3], [35] in murine T cells by Western blotting. Therefore, while *dlg4* may play a role in T cells, our studies focused on *dlg1* which has been implicated in several key aspects of T cell function [3], [7], [22]–[24], [26], [28], [36].

### The Generation of *dlg1* Germline and Conditional Knockout Mice Using Three Independent Approaches

To address the role of Dlg1 in T cell development, three independent *dlg1* knockout mouse models were generated; two models contained a germline deletion in *dlg1* and one model was generated with a conditional loss of *dlg1* confined to the T cell compartment.

**Figure 1:**
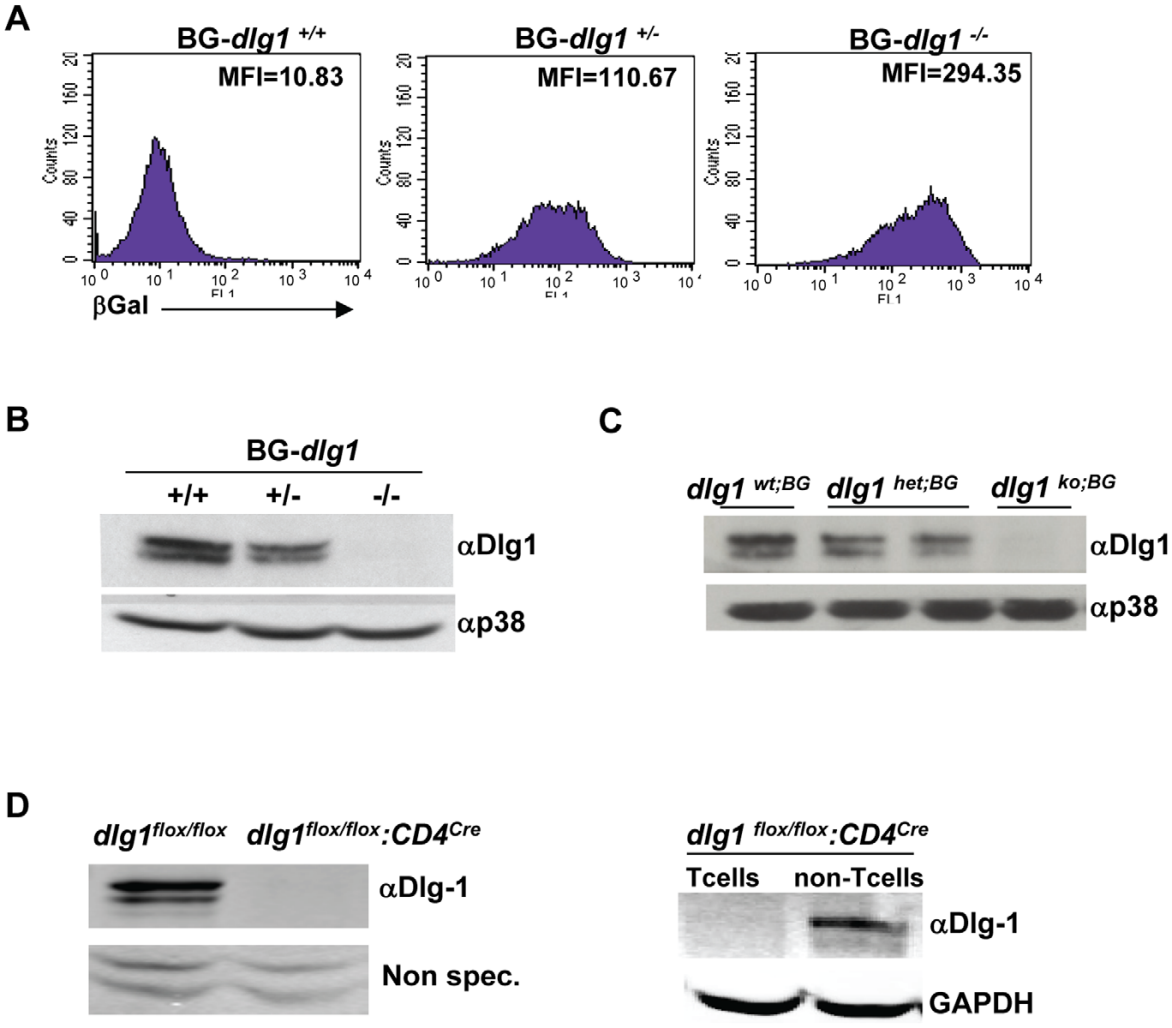
Dlg1 expression is ablated in the *dlg1^ko;BG^* and *dlg1^flox/flox^:CD4^cre^* mouse models. (A) Representative flow cytometry histograms show differential β-gal levels in fetal liver cells from BG *dlg1^+/+^* (*left*), *BG dlg1^+/−^* (*center*), or BG *dlg1^−/−^* (*right*) pups (n = 10 independent experiments). (B) Representative western blot of Dlg1 expression in whole cell lysates from BG *dlg1^+/+^*, BG *dlg1^+/−^,* or BG *dlg1^−/−^* fetal tissue using antibodies against Dlg1 or p38 (n = 6 independent experiments). (C) Western blot demonstrating Dlg1 expression in total splenocytes obtained from *dlg1^wt;BG^* and *dlg1^ko;BG^* mice 8 weeks post adoptive transfer. Data are representative of 3 independent experiments (D) *Left*, lymphocytes from Dlg1^flox/flox^ or Dlg1^flox/flox^:CD4^cre^ mice were enriched for T cells and whole cell lysates immunoblotted with antibodies against Dlg1. Non-specific bands (Non-spec.) from the same gel demonstrate equivalent loading in each lane. *Right*, splenocytes from Dlg1^flox/flox^:CD4^cre^ mice were enriched or depleted for T cells, and lysates were immunoblotted with antibodies against Dlg1 or GAPDH, as indicated.

Since mice that are germline deficient for *dlg1* develop abnormally and exhibit perinatal lethality due to cleft palate and the inability to suckle [37], both mouse models containing a *dlg1* germline deletion were maintained and bred as heterozygotes. Resulting embryos were then used as donors for partial or full hematopoietic reconstitution of irradiated recipient mice, as indicated below, to evaluate *dlg1-*dependent T cell development and function. In one approach, mice were generated with a tissue-wide knockout of *dlg1* using RRN196 ES cells generated by the Bay Genomics (BG) Consortium, to yield BG-*dlg1* ^−/−^mice (Miceli group). In this construct, the insertional mutation occurs in the fourth exon of *dlg1,* resulting in the first 150 amino acids of Dlg1 being fused to a β-galactosidase (β-Geo) insertion cassette (Figure S2A). While BG-*dlg1* ^+/+^, ^+/−^, and ^−/−^ pups were present at the expected Mendelian ratios, BG-*dlg1* ^−/−^ pups died shortly after birth, as expected, from severe birth defects. T cell development and function were therefore evaluated by adoptively transferring fetal liver cells from BG-*dlg1* ^+/+^and ^−/−^ donor littermates into sublethally irradiated Rag1 deficient mice (*rag1^−/−^*) and will herein be referred to as *dlg1^wt;BG^* or *dlg1^ko;BG^* mice, respectively. Briefly, BG-*dlg1* heterozygote mice were bred and fetal livers harvested from embyros at embryonic day 14.5. Prior to injecting fetal liver cells into recipient mice, a FACS-based β-galactosidase assay was used as a preliminary screen to differentiate between wildtype, heterozygous, and homozygous BG-*dlg1* donors (Figure 1A). Donor genotypes were confirmed by PCR using primer sets spanning exon 4 and the β-Geo gene to determine the presence and/or absence of the β-Geo insertion cassette (Figure S2C). Western blotting of fetal tissue confirmed the absence of Dlg1 protein expression in BG-*dlg1* ^−/−^ donor pups (Figure 1B) as well as in lymphoid organs from *dlg1^ko;BG^* mice 8 weeks post-adoptive transfer (Figure 1C).

A second approach utilized a previously published *dlg1* germline deficient mouse generated via gene trap (GT) insertion (Russell group) [37]. In this construct, the first 549 amino acids were fused to a β-galactosidase insertion cassette, resulting in a tissue-wide loss of *dlg1* expression (Figure S2B). *dlg1*-deficientT cells were generated by hematopoietic reconstitution of lethally irradiated B6-Ptprca (Ly5.1) mice using fetal liver cells from GT-*dlg1^−/−^* gene trap mice or wild-type littermates and will herein be referred to as *dlg1^ko;GT^* or *dlg1^wt;GT^* mice, respectively.

In a third approach, the role of *dlg1* in T-lineage cell development was directly addressed by generating a conditional knock out mouse (Burkhardt group). Mice bearing a loxP-Dlg1 targeting allele, *dlg1^flox/flox^* [38], were crossed to CD4-Cre transgenic mice, in which Cre recombinase is driven by the CD4 promoter so that gene knockout is induced during the CD4^+^CD8^+^ double positive stage of T cell development. Resulting progeny with a targeted deletion of *dlg1* late in thymic development will herein be referred to as *dlg1^flox/flox^*:CD4*^cre^*. *dlg1^flox/flox^*:CD4*^cre^* mice were viable and born in normal Mendelian ratios with no gross morphological defects (data not shown). While T cells from *dlg1^flox/flox^* mice had detectable levels of Dlg1 protein, following Cre-mediated deletion T cells from *dlg1^flox/flox^*:CD4*^cre^* lacked detectable Dlg1 protein (Figure 1D, left panel). Importantly, the *dlg1* deletion was restricted to the T cell compartment, as T-depleted lymphocytes from *dlg1^flox/flox^*:CD4*^cre^* transgenic mice maintained Dlg1 expression (Figure 1D, right panel). Mice with or without a conditional loss of *dlg1* in the T cell compartment will herein be referred to as *dlg1^flox/flox^*:CD4*^cre^* and *dlg1^flox/flox^* mice, respectively.

### Lymphocyte Development Appears Normal in *dlg1* Knock-out Mice

**Figure 2:**
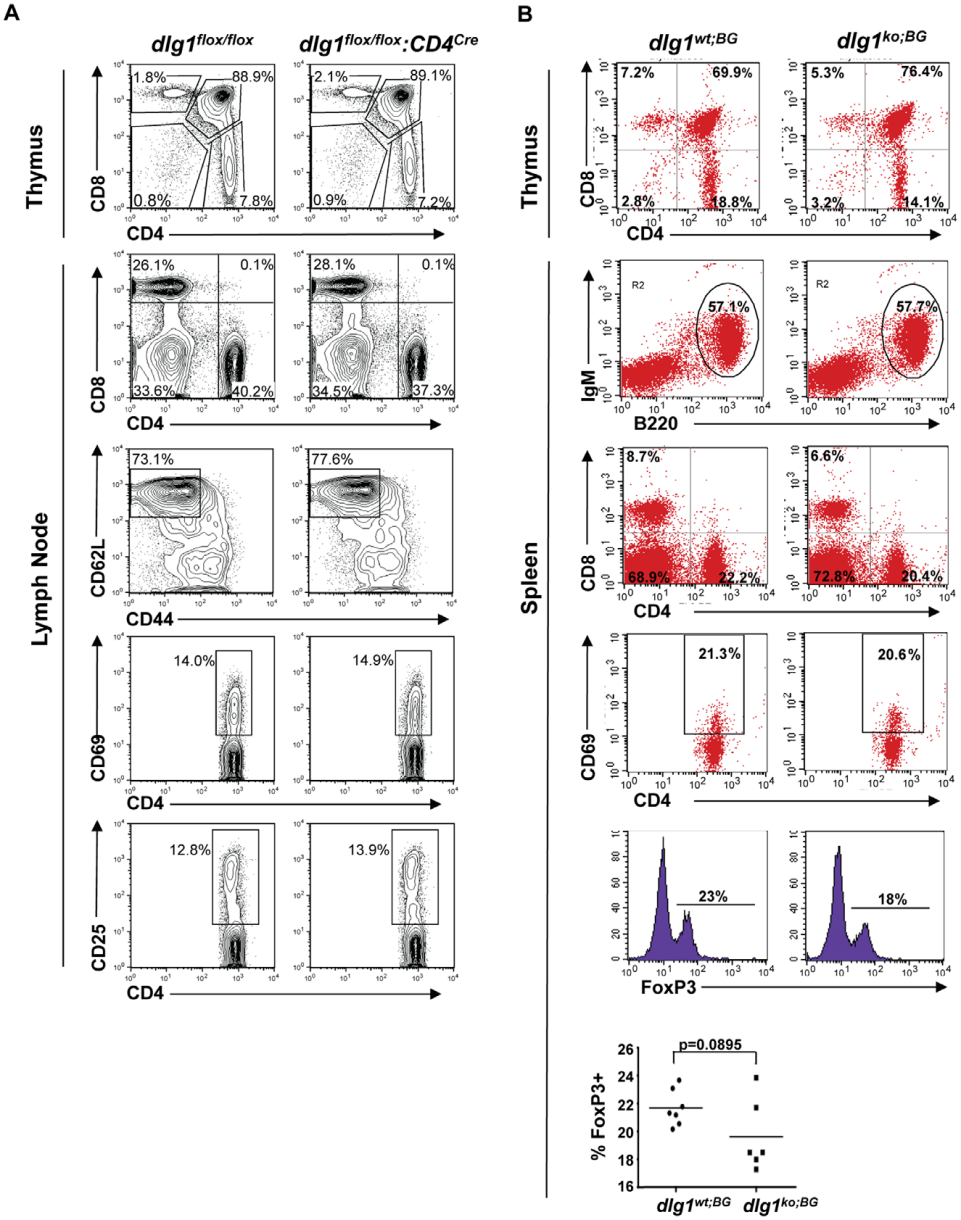
Lymphocyte development is not affected in *dlg1* knockout mice. (A) Flow cytometry profiles of thymocytes (Thymus) and lymph node cells (Lymph Node) from *dlg1^flox/flox^* or *dlg1^flox/flox^:CD4^cre^* mice stained with the indicated antibodies. (B) Flow cytometry profiles of thymocytes (Thymus) and splenocytes (Spleen) from *dlg1^wt;BG^* or *dlg1^ko;BG^* mice stained with the indicated antibodies. Splenic Treg populations were determined by dual CD4+ surface and FoxP3 intracellular staining. Data are representative of 3 independent adoptive transfer experiments, where n≥6 for each genotype.

Previous studies have demonstrated a role for Dlg1 in regulating the activation of NFAT [1], [23], [24], [26], [28], a key transcription factor in T and B cell development and activation. To examine the role of *dlg1* ablation in the development of T cells and other lymphocyte populations, thymic and peripheral lymphocyte subsets were analyzed from *dlg1^wt;BG^* vs. *dlg1^ko;BG^, dlg1^wt;GT^* vs. *dlg1^ko;GT^*,and *dlg1^flox/flox^* vs *dlg1^flox/flox^:CD4^cre^* mice. Examination of the lymphoid organs from all three *dlg1* knockout mice revealed no significant difference in total cellularity in the spleen, thymus, or lymph nodes compared to their respective wild-type littermates (Table S1 and data not shown). In addition, reconstitution was found to be equally efficient in *dlg1^wt;GT^* and *dlg1^ko;GT^* mice as measured by comparing CD45.1 with CD45.2 (data not shown) and by cell counts for various peripheral blood cell and bone marrow populations in both *dlg1^ko;GT^* and *dlg1^ko;BG^* mice (Figure S3 and data not shown). Analysis of developing T cell populations from the thymus revealed no developmental blocks in any of the three mouse models assessed (Figure 2A and B, upper panels, Table S1), and comparable B cell (IgM^+^B220^+^) and T cell populations, including naïve T cells (CD62L^high^CD44^lo^), were found in the periphery. CD4^+^ vs CD8^+^ ratios and CD69^+^ T cell subsets also appeared normal (Figure 2A and B, Table S1, and data not shown). Notably, while (CD4^+^CD25^+^) regulatory T cell subsets appeared unremarkable in *dlg1^flox/flox^:CD4^cre^* and *dlg1^ko;GT^* mice, *dlg1^ko;BG^* mice exhibited a trend towards a modest reduction in CD4^+^FoxP3^+^ splenic cells in 2 out of 3 experiments (2 experiments, WT n = 7, KO n = 6) both by percentage and cell number (Figure 2B, bottom panels and data not shown). These data suggest that although *dlg1* appears to be dispensable for T cell development in the context of these mouse models, it may contribute to the development of Treg populations.

### TCR-induced Regulation of Early T Cell Activation Makers is Intact in *dlg1* Knockout Mice

**Figure 3:**
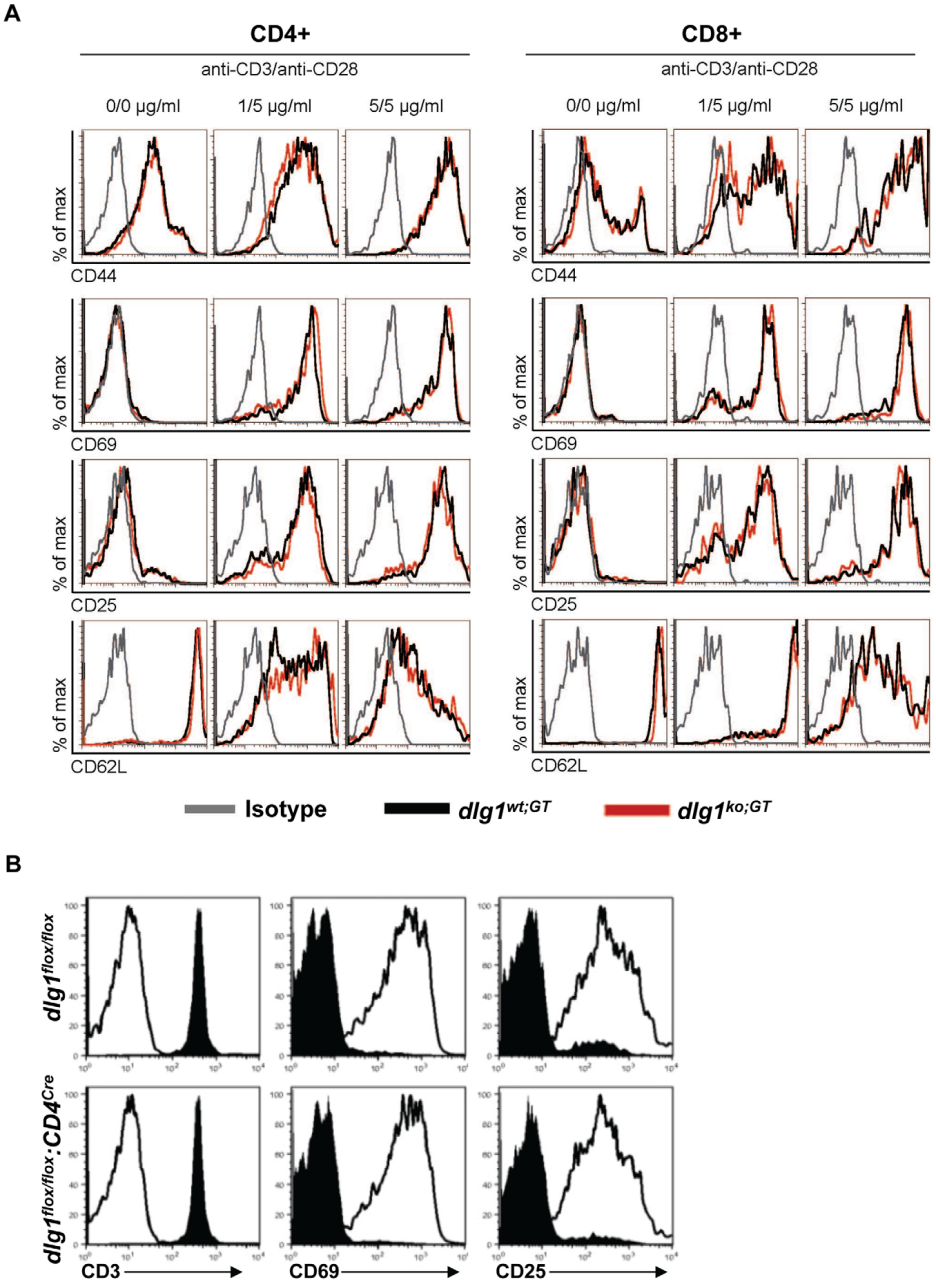
The regulation of T cell surface activation markers is unaffected in *dlg1* knockout mice. (A) Splenocytes isolated from *dlg1^wt;GT^* (black) or *dlg1^ko;GT^* (red) mice were activated with indicated concentrations of plate bound anti-CD3 in the presence of anti-CD28 for 24 hours and assessed for the expression of activation markers. Histograms were gated on viable donor derived (CD45.2^+^) CD4^+^ or CD8^+^ T cells and show one representative mouse per genotype (n≥3). (B) Flow cytometry profiles of selected surface markers on purified CD4^+^ T cells from *dlg1^flox/flox^* (top *panel*) or *dlg1^flox/flox^:CD4^cre^* (*bottom panel*) mice stimulated with T-depleted splenocytes with (unshaded) or without (shaded) anti-CD3 antibody for 24 hrs (n≥3).

To examine if T cells from *dlg1* knockout mice could properly modulate the expression levels of defined cell surface activation markers in response to TCR-mediated signals, naïve splenocytes from *dlg1^ko;GT^* and CD4^+^ T cells from *dlg1^flox/flox^:CD4^cre^* mice were stimulated with anti-CD3/CD28 or anti-CD3, respectively. T cells from both germline and conditional *dlg1* knockout mice were found to up-regulate early activation markers, including CD25 and CD69 (Figure 3A and B), and down-regulate CD62L (Figure 3A) or CD3 (Figure 3B) expression to a level comparable to that found on wild-type T cells. These data indicate that *dlg1* is not required for TCR-dependent regulation of early cell surface activation markers in these mouse models.

### TCR-dependent Pan Tyrosine- and Alternative p38- Phosphorylation are Unaffected in *dlg1* Knockout Derived T Cells

**Figure 4:**
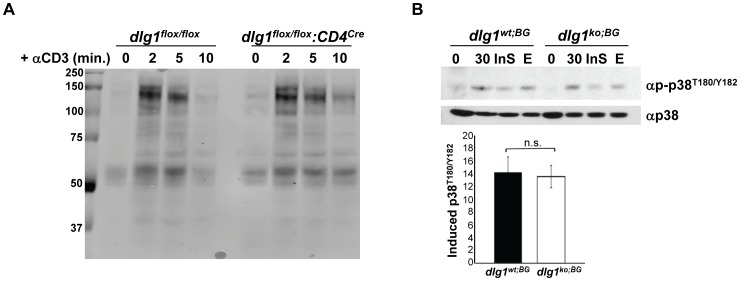
T cells from *dlg1* knockout mice show normal TCR-induced tyrosine- and alternative p38- phosphorylation. (A) Purified CD4^+^ T cells from *dlg1^flox/flox^* or *dlg1^flox/flox^:CD4^cre^* mice were stimulated with anti-CD3 antibody for the indicated times. Whole cell lysates were immunoblotted with anti-phosphotyrosine antibody. (B) *(Top panel)* Expanded T cells from *dlg1^wt;BG^* or *dlg1^ko;BG^* mice were restimulated with anti-CD3 and anti-CD28 antibodies for 30 minutes in the absence or presence of an Insolution p38 (InS) or U0126 Erk (E) inhibitor. Whole cell lysates were then immunoblotted with anti-phospho-p38 (T180/Y182) followed by anti-p38 to assess loading. *(Bottom panel)* Levels of induced p38 phosphorylation relative to corresponding unstimulated samples were determined by densitometry and normalized according to loading controls (n = 3 each for *dlg1^wt;BG^* and *dlg1^ko;BG^*). Data represent mean +/− StDev. n.s. = not significant, (p = 0.7236).

Dlg1 has been shown to orchestrate TCR proximal signaling by facilitating interactions between multiple kinases and effector proteins [22], however examination of TCR-induced total tyrosine phosphorylation revealed no gross changes in the pattern of phosphoproteins between *dlg1^flox/flox^* and *dlg1^flox/flox^:CD4^cre^* CD4^+^ T cells nor between T cells obtained from *dlg1^wt;BG^* and *dlg1^ko;BG^* mice (Figure 4A and data not shown). Strikingly, alternative p38 phosphorylation, as measured by specific dual-phosphorylation at residues T180 and Y182, also appeared unaffected in *dlg1^ko;BG^* T cells (Figure 4B). This is in contrast to previous studies utilizing acute knockdown, which demonstrated a pivotal role for Dlg1 in mediating alternative p38 phosphorylation and activation [24], [26]. These data indicate that TCR-mediated proximal signaling events, including alternative p38 phosphorylation, are not affected in T cells from *dlg1^flox/flox^:CD4^cre^* and *dlg1^ko;BG^* mice.

### TCR-induced Actin Polymerization is Defective in T Cells with Acute, but not Germline or Conditional, *dlg1* Ablation

**Figure 5:**
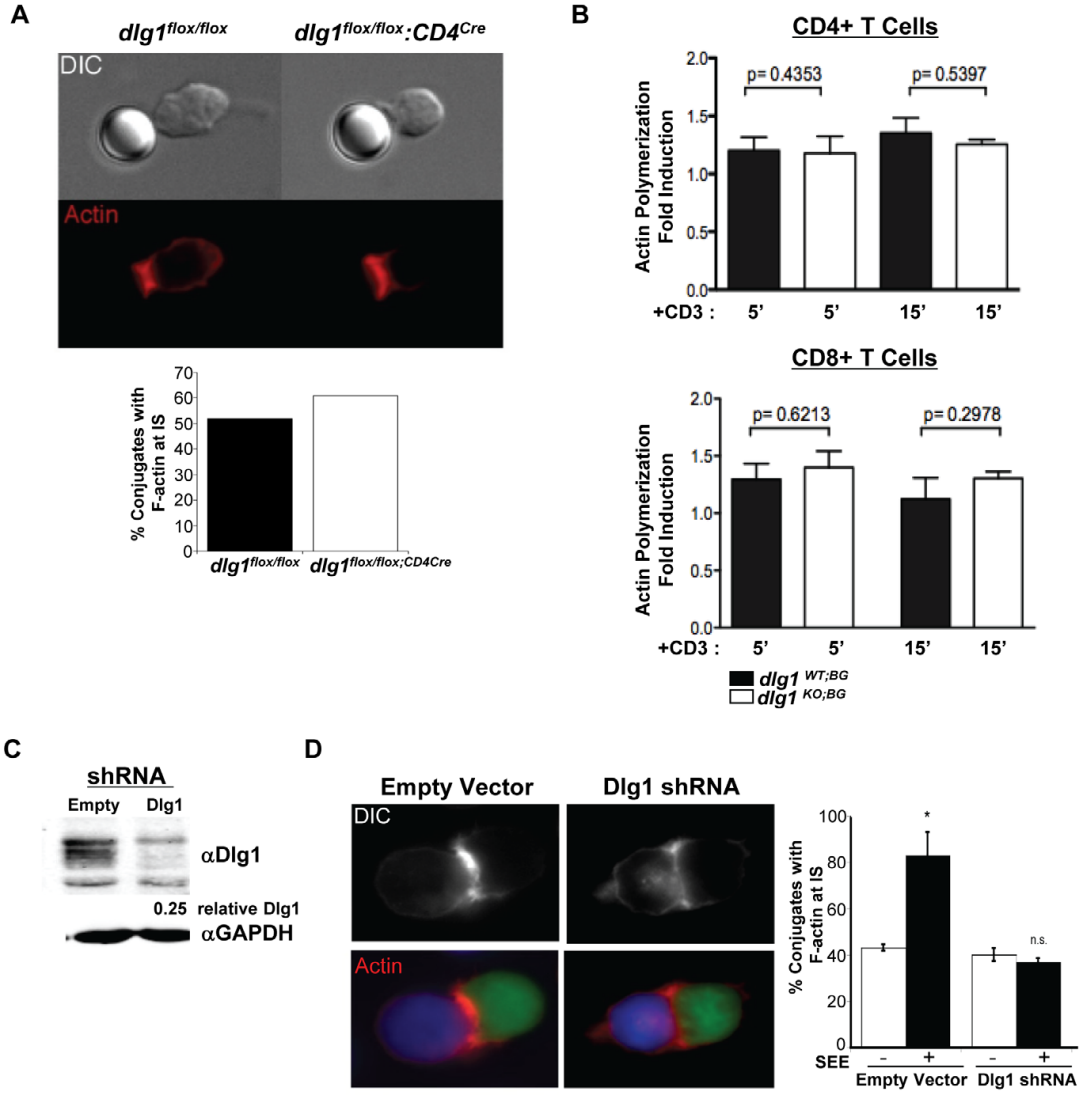
An acute, but not germline or conditional, loss of *dlg1* impairs receptor-mediated actin polymerization. (A) *dlg1^flox/flox^* or *dlg1^flox/flox^:CD4^cre^* CD4^+^ T cells were allowed to conjugate with anti-TCR antibody-coated beads for 20 minutes and stained for actin with rhodamine-phalloidin (*top panel*). At least 50 cells were scored for actin localization to the T cell/bead interface (*bottom panel*). (B) Expanded T cells from *dlg1^wt;BG^* or *dlg1^ko;BG^* mice were restimulated with plate-bound anti-CD3 and anti-CD28 antibodies for 5 or 15 minutes. Cells were stained with a combination of anti-CD4, anti-CD8, and FITC-phalloidin and assessed by FACS to determine the relative level of induced actin polymerization in CD4^+^ (*top panel*) and CD8^+^ (*bottom panel*) T cell populations. These data are representative of 2 independent experiments, n≥4 for WT and KO samples. (C) Whole cell lysates from Jurkat cells expressing empty vector (empty) or Dlg1 shRNA were immunoblotted with antibodies against Dlg1 or GAPDH, as indicated. (D) Jurkat T cells (green) were transfected with either pCMS3.eGFP.H1p empty vector or pCMS3.eGFP.H1p containing a shDlg1 target sequence. Cells were stimulated with SEE-pulsed (or untreated) Raji B cells (blue) and stained with rhodamine-phalloidin (red) (*left panel*). At least 50 conjugates were scored for F-actin localization to the T cell/APC interface in each of two independent experiments (*right panel*). Data represent mean +/− StDev. n.s., not statistically significant.

WASp/WAVE family members function to mediate changes in the actin-cytoskeleton through the formation of F-actin [2], [4]. Previously Dlg1 had been shown to interact with WASp and to play a role in orchestrating TCR-induced actin polymerization and polarized synaptic raft clustering in the context of shRNA-mediated Dlg1 knockdown in CD8+ T cells [22], [24]. To assess actin polymerization at the IS in the context of total *dlg1* ablation, *dlg1^flox/flox^* ^;^ and *dlg1^flox/flox^:CD4^cre^* CD4+ T cells were allowed to conjugate with anti-TCR antibody-coated beads for 20 minutes and scored for actin localization to the T cell/bead interface (Figure 5A). In addition, *dlg1^ko;BG^* derived T cells were assessed for TCR-induced actin polymerization by flow cytometry at various time points (Figure 5B). In both experiments, no significant defects were observed in TCR-induced actin polymerization in T cells derived from either *dlg1^flox/flox^:CD4^cre^* or *dlg1^ko;BG^* as compared with their wild-type counterparts suggesting *dlg1* is not required for TCR-induced actin polymerization in these mice.

This outcome contrasts with previous findings from the Miceli lab showing that Dlg1 knockdown impairs TCR actin polymerization in primary murine T cells [22]. We therefore tested actin polymerization in the context of acute Dlg1 deletion in an independent experimental system. Dlg1 suppressed Jurkat T cells were generated using an shRNA based vector. After optimization, Dlg1 expression in cells transfected with Dlg1 shRNA could typically be reduced to ∼25% of control levels (vector alone), as judged by Western blot analysis (Figure 5C). Suppression to at least 25% of control was confirmed for all functional studies. Jurkat T cells transfected with control or Dlg1 shRNA were then conjugated to SEE-pulsed Raji B cells 72 hours post-transfection, and actin responses at the IS were analyzed by fluorescence microscopy. No gross changes were observed in the efficiency of SEE-induced conjugate formation as a result of Dlg1 suppression. Nonetheless, in contrast to T cells with a conditional or germline genomic deletion, T cells with acute Dlg1 suppression exhibited diminished F-actin accumulation with fewer Dlg1-deficient T cells accumulating F-actin at the IS (Figure 5D). These data are consistent with previous experiments performed in primary murine T cells [22]. We conclude that the effects on T cell actin responses differ in acute vs. long term models of *dlg1* deficiency, possibly because compensatory changes in the long-term knockout models mask the contribution of *dlg1*.

### Polarization and Migration is not Affected in Activated T Cells from Dlg1 Knockout Mice

**Figure 6:**
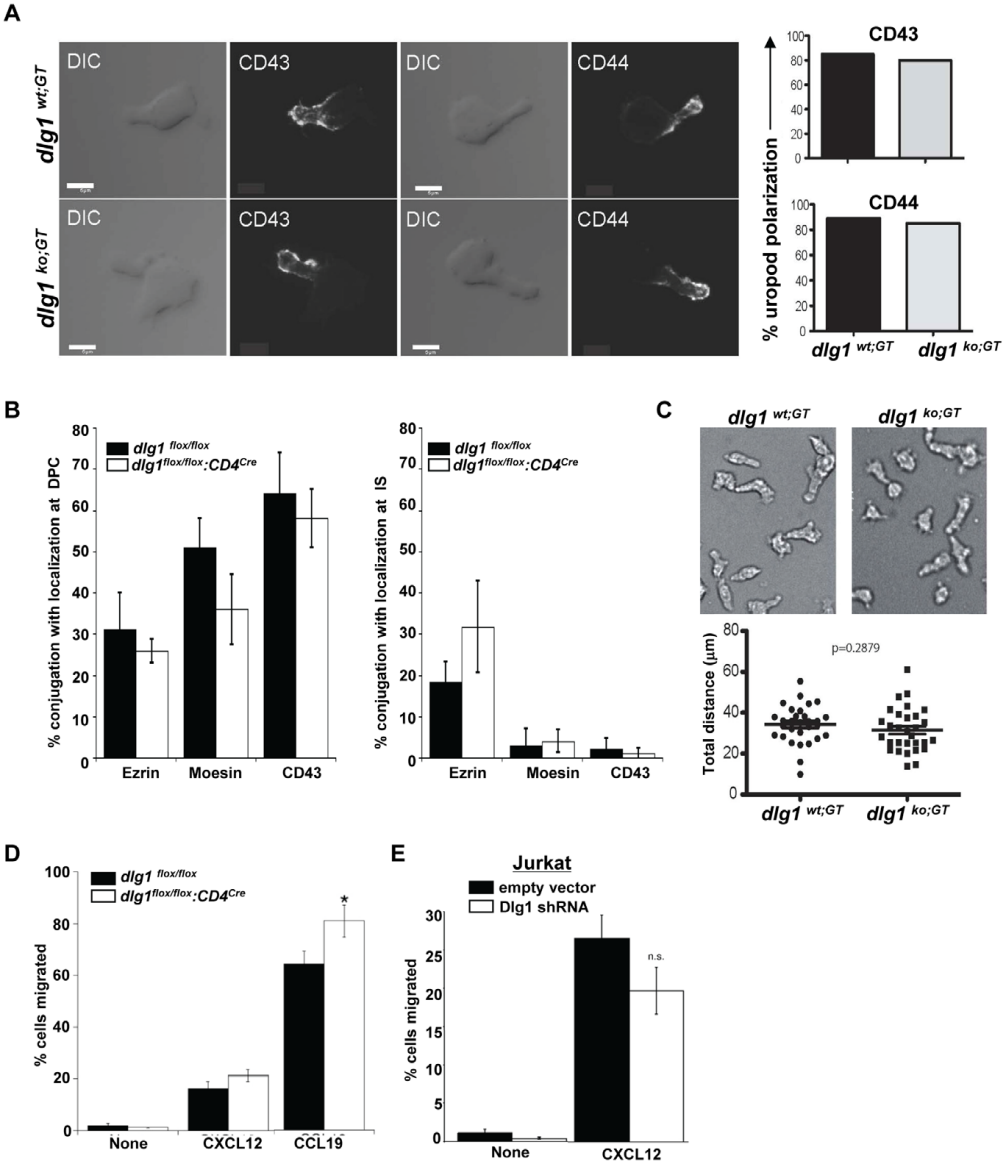
T cell polarization and migration are not hindered by the loss of *dlg1*. (A) *dlg1^wt;GT^* or *dlg1^ko;GT^* activated T lymphoblasts were stained with CD43 or CD44 and assessed for protein polarization (*left panel*). The percentage polarization of CD43 and CD44 to the T cell uropod was determined by scoring 45 cells in two independent experiments (*right panel, top and bottom*). (B) *dlg1^flox/flox^ or dlg1^flox/flox^:CD4^cre^* CD4^+^ T cells were allowed to conjugate with anti-TCR antibody coated beads for 20 minutes, fixed and stained with the indicated antibodies. The indicated proteins were scored for localization to the DPC (*left panel*) or IS (*right panel*). 50–100 conjugates were scored for F-actin localization to the T cell/APC interface in each of 2–3 independent experiments (*right panel*). Data represent mean +/− StDev. Differences were not statistically significant for any sample pair. (C) *dlg1^wt;GT^* or *dlg1^ko;GT^* activated T lymphoblasts were subjected to time-lapse microscopy and assessed for random migration (*top panels*). Total distance was determined from DIC images acquired at 1 min intervals by tracking a total of 30 cells over a 30 min period (*bottom panels*). Data are representative of n = 2 experiments. (D) A modified Boyden chamber was used to assess the percent of *dlg1^flox/flox^* or *dlg1^flox/flox^:CD4^cre^* T cells which migrated in response to no chemokine, CXCL12, or CCL19 over 2 hours and was calculated as the ratio of the total cells, to cells that migrated * = p≤0.05. (E) The percentage of Jurkat cells transfected with either empty vector or shDlg1 which migrated in response to no chemokine or CXCL12 for 2 hours was calculated as: number of cells migrated/total number of cells. n.s. = no significiant difference.

The formation of the IS results in the asymmetric distribution of select proteins to the IS and DPC [3], [7]. Furthermore, the acute loss of Dlg1 and its functional partner Scribble in a T cell line has been shown to disrupt both random migration and uropod formation [3]. Therefore, activated T cells deficient in Dlg1 were assessed for morphology and polarization by examining the localization of specific surface and intracellular protein markers on in vitro cultured cells. *dlg1^ko;GT^* cells displayed normal uropod formation as well as normal polarization of the uropod markers CD43 and CD44 (Figure 6A).

Similarly, comparison of control and *dlg1^flox/flox^:CD4^cre^* derived CD4+ T cells following conjugation with anti-TCR antibody-coated beads showed no significant differences in the localization of three well-defined marker proteins (Figure 6B). As reported previously, ezrin localizes to both the IS and the DPC [30], while moesin and CD43 localize to the DPC. PKCζ also polarized efficiently to the DPC in these cells (data not shown).

To examine the role of *dlg1* in random T cell migration, T cells which had been pre-activated with Concanavalin A for 5 days from *dlg1^wt;GT^* and *dlg1^ko;GT^* mice were compared for their ability to migrate using time-lapse microscopy. However no significant difference was observed in the distance traveled between *dlg1^wt;GT^* and *dlg1^ko;GT^* T cells (Figure 6C).

The role of *dlg1* in chemokine-directed migration was examined in both *dlg1^flox/flox^:CD4^cre^* CD4^+^ and Dlg1-suppressed Jurkat T cells. Despite the diminished actin polymerization observed in T cells expressing Dlg1 shRNA, no reduction was observed in the migration of control cells and *dlg1^flox/flox^:CD4^cre^* CD4^+^ T cells, or between Dlg1-suppressed and control Jurkat T cells in response to chemokines (Figure 6D and E). A modest, but statistically significant, increase was detected in the migration of *dlg1^flox/flox^:CD4^cre^* CD4^+^ T cells toward CCL19. The significance of this is unclear however, as no increase was observed in response to CXCL12. Nonetheless, these data suggest that *dlg1* may be dispensable for chemokine-induced actin-mediated motility.

### TCR-induced Proliferation is not Affected in T Cells Derived from *dlg1* Germline Knockout Mice

**Figure 7:**
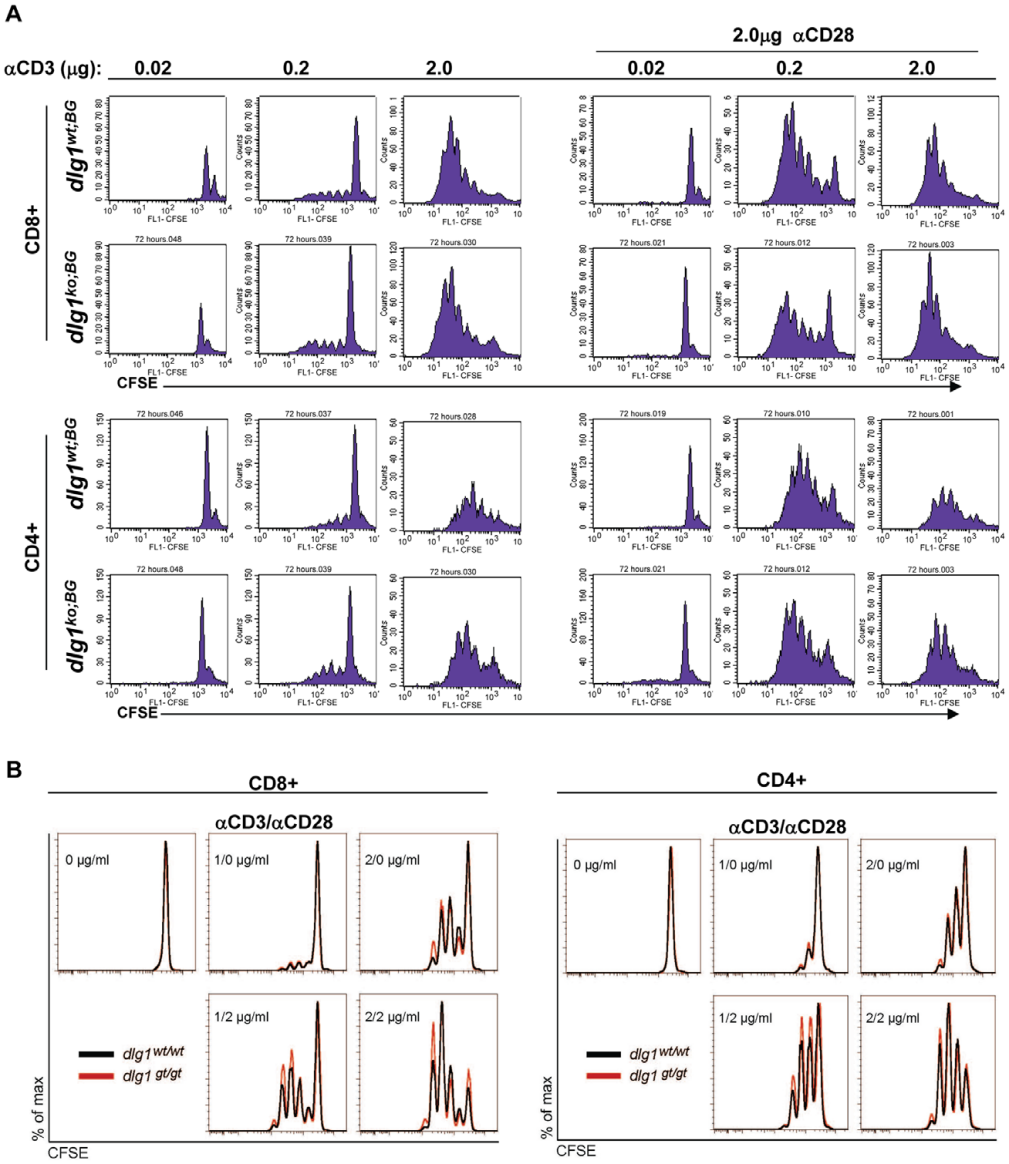
T cells from *dlg1* germline knockout mice proliferate comparably to wildtype T cells. (A) *dlg1^wt;BG^* or *dlg1^ko;BG^* derived splenocytes were stained with CFSE and stimulated with various concentrations of anti-CD3 alone or anti-CD3 and anti-CD28 antibodies for 72 hours. Cells were subsequently surface stained with anti-CD8 or anti-CD4 antibodies and T cell populations analyzed by flow cytometry. (B) Naive splenic T lymphocytes isolated from *dlg1^wt;GT^* (black) or *dlg1^ko;GT^* (red) were labeled with CFSE and activated with the indicated concentrations of plate bound anti-CD3 in the presence or absence of anti-CD28. CFSE profiles at 62 hours were gated on CD8^+^ or CD4^+^ T cells and are representative of 2 independent experiments.

The Drosophila ortholog of *dlg1* was originally identified as a tumor suppressor and attenuator of cell division [21]; thus alteration of its expression may affect cellular proliferation rates. Further, *dlg1* knockout has been reported to promote TCR-induced proliferation in naïve CD4+ and CD8+ T cells [36]. To address the role of *dlg1* in TCR-dependent proliferation in our models, naïve splenocytes or lymph node cells from *dlg1^wt;BG^* and *dlg1^ko;BG^* were labeled with CFSE and stimulated for 24, 48, or 72 hours with varying concentrations of anti-CD3 alone or in combination with anti-CD28 (Figure 7A and data not shown). In addition, naïve splenocytes from *dlg1^wt;BG^* and *dlg1^ko;BG^* were enriched for CD8^+^ T cells and their proliferative response assessed by ^3^H-Thymidine incorporation (data not shown). In both assays, the rates of TCR-mediated proliferation were indistinguishable between *dlg1^wt;BG^* and *dlg1^ko;BG^* derived T cells at all antibody concentrations and time points assessed. Similarly, no differences were observed in the proliferative responses between *dlg1^wt;GT^* and *dlg1^ko;GT^* derived CD4+ and CD8+ T cells in the presence of varying concentrations of anti-CD3 alone or in combination with anti-CD28 as measured by CFSE dilution (Figure 7B). These results were in contrast to previously published work by Stephenson et al. which indicated that *dlg1* knockout leads to hyper-proliferation of naïve CD4 and CD8 T cells in response to TCR/CD28 stimulation [36]. While our data suggest that *dlg1* does not function to regulate receptor-mediated proliferation in T lymphocytes in our mouse models, it remains possible that compensatory mechanisms may have masked a measurable phenotype.

### Th1/Th2 Type Cytokine Production is Affected by the Acute or Conditional, but not Germline, Loss of *dlg1*

**Figure 8:**
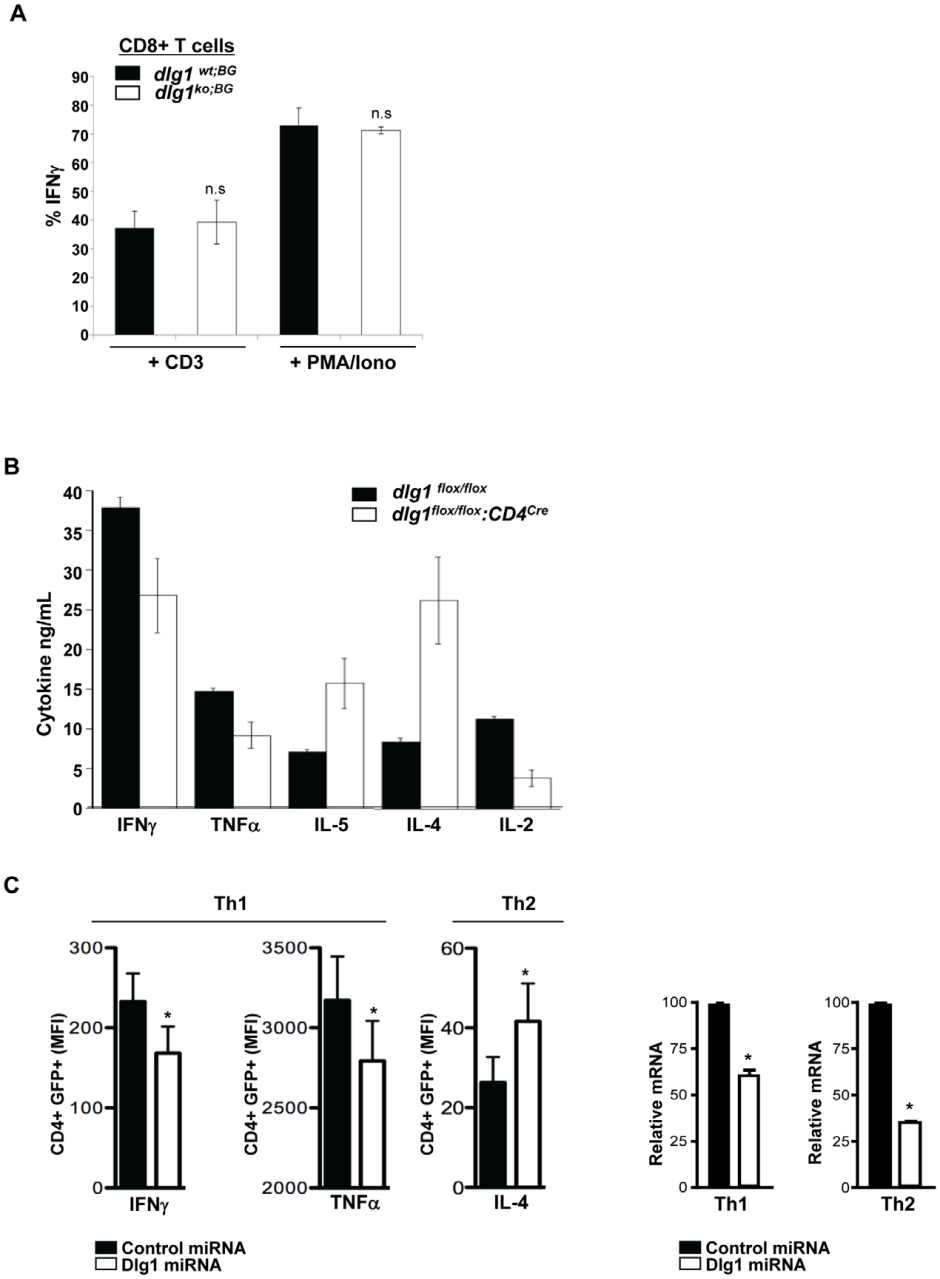
The acute or conditional loss of *dlg1* results in differential Th1/Th2-type cytokine production. (A) Expanded splenocytes from *dlg1^wt;BG^* or *dlg1^ko;BG^* mice were restimulated with plate-bound anti-CD3 and anti-CD28 antibodies or PMA/Ionomycin for 4 hours. Cells were stained with anti-CD8, permeabilized, and subsequently stained with anti-IFN-γ to determine intracellular cytokine levels (n≥3 each for *dlg1^wt;BG^* and *dlg1^ko;BG^* mice). Data represent mean +/− StDev. n.s. = no significance where p>0.05. (B) Expanded CD4^+^ T cells from *dlg1^flox/flox^* or *dlg1^flox/flox^:CD4^cre^* mice were restimulated with plate-bound anti-CD3 and anti-CD28 antibodies for 24 hours. Cell supernatants were collected and cytokine production analyzed by ELISA. Standard deviations were calculated from triplicate stimulations and statistical significance determined using a paired Student’s t test. *p<0.05. (C) Th1 or Th2 polarized T cells from C57Bl/6 mice were transduced with either control- or Dlg1-miRNA retrovirus (*right panel*) and subsequently stimulated with plate-bound anti-CD3/CD28 for 6 hours. Cells were then surface stained with anti-CD4 followed by intracellular staining with antibodies against either IFNγ or TNFα (Th1 cells) or IL-4 (Th2 cells). Cells were analyzed by FACS and gated on CD4+ and GFP+ (miRNA vector) cells to determine the relative level of cytokine production (MFI) in control and knockdown populations (*left panel*) (IFNγ, TNFα n = 4; IL-4 n = 3). Data represent means from ≥3 experiments ± SEM. *p<0.05.

The differential reorganization of T cell membrane proteins and intracellular complexes during IS and DPC formation has been shown to modulate effector cytokine production [13], [15]. To evaluate the effect of *dlg1* genomic ablation on TCR-induced effector cytokine production, *dlg1^wt;BG^* and *dlg1^ko;BG^* CD8^+^ expanded T cells were assessed following 4 hours of stimulation with plate-bound anti-CD3. FACS analysis of intracellular protein levels revealed no significant differences in TCR-induced IFNγ or IL-2 production between CD8+ T cells from *dlg1^wt;BG^* and *dlg1^ko;BG^* mice (Figure 8A and data not shown). These results were in contrast to previous experiments in which CD8+ T cells with a transient Dlg1 deficiency via knock-down exhibited diminished IFNγ and IL-2 cytokine production in response to TCR engagement compared to wild-type cells [22], [24]. Notably, purified, expanded CD4^+^ T cells from *dlg1^flox/flox^* and *dlg1^flox/flox^:CD4^cre^* mice that were re-stimulated with anti-CD3 demonstrated that *dlg1^flox/flox^:CD4^cre^* CD4^+^ T cells produced less IL-2 and Th1-associated cytokines IFNγ and TNFα, while levels of the Th2-associated cytokines IL-4 and IL-5 were significantly increased in comparison to wild-type littermates (Figure 8B).

To further examine the effect of acute Dlg1 diminution on Th-type cytokine production, Th1 and Th2 polarized cells from wild-type C57Bl/6 mice were transduced with either control- or Dlg1-miRNA expressing retroviruses resulting in an approximately 50% reduction of Dlg1 expression (Figure 8C, far right panels). Similar to *dlg1^flox/flox;CD4cre^*CD4+ T cells, restimulation of Dlg1-suppressed Th1 cells resulted in significantly diminished levels of intracellular IFNγ and TNFα , whereas Dlg1 suppressed Th2 cells demonstrated significantly enhanced IL-4 production (Figure 8C, left panels). In combination, these data suggest that *dlg1* may play differential roles in the Th response; helping to promote Th1 responses, while suppressing Th2 responses.

## Discussion

Polarity proteins have recently been implicated as key regulators of T cell migration, signaling, effector function, and fate [3], [12], [14]. Dlg1, a member of the ancestral Scribble polarity network, directly binds and regulates the activity of the proximal TCR-associated kinases and has been implicated in regulating TCR-mediated signal transduction and cell polarity in the context of IS formation and cell migration. Although studies using Dlg1 knockdown or over-expression technologies in primary T cells and transformed cell lines have demonstrated a role for Dlg1 in T cell signaling, activation, and the establishment of synaptic polarity during antigen recognition [22]–[24], its function in the context of T cell development, immune homeostasis or immune response *in vivo* remains unclear. This is due in part to seemingly conflicting reports which have alternatively suggested that Dlg1 is a negative regulator [23], [36], or a positive regulator [22], [24], [26], [28], [30] of T cell function.

In this study, three independent *dlg1*-deficient mouse models were generated and examined in an effort to unravel the functional significance of Dlg1 in T cell development and effector function in a null background. In general, we found that germline and conditional *dlg1* knockout mice had no apparent defects in CD4+ or CD8+ T cell polarization-dependent events such as development, migration, activation, signaling or proliferation. These data were surprising considering previous reports demonstrating a role for Dlg1 in regulating TCR-mediated actin polymerization, signal specificity and function, in the context of acute knockdown or over-expression in mature T cells. While we observed diminished cytoskeletal reorganization in primary mouse CD8+ [24] and human Jurkat T cells (Figure 5D) with acute Dlg1 knockdown, we did not observe defects in mature T cells from conditional or germline *dlg1* knockout mouse models (Figure 5A and B). These data suggest that the timing and/or duration of *dlg1* ablation may greatly affect the phenotype of Dlg1-deficient cells and impact the ability to examine the role Dlg1 on T cell function. This hypothesis is supported by studies of Th1 and Th2-type cytokine secretion, which demonstrated differential cytokine secretion in T cells with an acute or conditional loss of Dlg1, but not a germline loss of *dlg1* (Figure 8). Because the conditional *dlg1* knockout leads to Dlg1 ablation later in T cell development, we hypothesize that a shorter developmental window was available for selection of cells that have compensated for Dlg1 loss. Indeed, while all three models demonstrated modest, if any, defects, it is noteworthy that the most significant defects observed (i.e. in Th1 and Th2 development) occurred under the conditions where *dlg1* was ablated later in development or in experiments using acute knockdown strategies.

The phenomenon of compensatory factors that mask the effects of Dlg1 deficiency is not unprecedented. In neuronal cells, evidence suggests a key role for Dlg1 in regulating synaptic AMPA receptor trafficking. However neurons from mouse embryos in which the *dlg1* gene has been ablated develop normally and form synapses with normal levels of AMPA receptors and no detectable abnormalities [39]. Investigators have hypothesized that the lack of a phenotype in Dlg1-mutant cells could be caused by the compensation of other Dlg-family members (Dlg2, Dlg3, Dlg4) or other adaptive processes during neuronal development and synapse maturation that could compensate for the normal function of Dlg1 [40]. Based on the results of our collaborative study, we hypothesize that similar event(s) could be occurring in developing T lymphocytes, making it difficult to appreciate the functional role of Dlg1 in developing and peripheral lymphocyte populations. Further support for this working hypothesis comes not only from comparative investigation within this study, but also from comparison of previously published works demonstrating a critical role for Dlg1 in cytoskeletal organization [24], [26], alternative p38 activation [24], [26], [28], NFAT activation [24], [26], [28] and effector function [24], [28], in the context of acute knockdown in T cells (Figures 5 and 6). An important area for future investigation will be to identify the changes in Dlg family protein expression and/or the regulation of other proteins that may compensate for the loss of Dlg1; this will lay the groundwork to more fully assess the significance of this pathway for T cell development and function.

Notably, our studies also uncovered novel contextual roles for Dlg1 as both a positive and negative regulator of T cell function, as Dlg1 ablation was found to inhibit Th1, while enhancing Th2, cytokine production in CD4+ T cells. Initial studies of Dlg1 by Xavier et al. found that the overexpression of Dlg1 in conjunction with Vav1, attenuated Vav1-induced NFAT activity in Jurkat T cells. These studies also found that the long term diminution of Dlg1 in Jurkat T cells via stable siRNA-based knockdown resulted in impaired NFAT reporter activation [23]. In a follow-up study, Stephenson et al provided data indicating that Dlg1 may negatively regulate T cell proliferation utilizing mouse *dlg1*-deficient T cells generated by recombination-activating gene 2 (*rag2*)-deficient complementation [36]. Both reports supported a role for Dlg1 as a negative regulator of T cell activation and function by suppressing NFAT-mediated transcription and cycle entry, respectively. However, two studies by Round et al., utilizing acute siRNA-based knockdown in mouse TCR transgenic CD8+ T cells found that Dlg1 knockdown attenuated NFAT-mediated transcription of endogenously regulated NFAT genes (NFATc1 and IFNγ), while complementary studies of Dlg1 overexpression resulted in a tightly regulated, dose-dependent enhancement of NFAT-mediated transcription. Moreover, Round et al provided clear biochemical evidence for a direct interaction between the MAP kinase p38 and Dlg1, which allows for nucleation of a signaling complex that results in alternative p38 activation and downstream NFAT phosphorylation [22], [24].

Collectively, these results may appear conflicting and contradictory, regarding a role for Dlg1 as a positive or negative regulator of T cell activation and function. However, our data supports a view where Dlg1 may specify TCR signals that enhance or attenuate particular T cell responses in a context dependent manner. Indeed, the differential assembly of membrane microdomains, signaling molecules, and co-polarization of cytokine receptors at the IS has been implicated in controlling memory versus effector cell development [20], as well as Th1/Th2 lineage commitment and effector function [15], [41], [42]. In this study, both conditional knockout and acute knockdown approaches demonstrated that Dlg1 positively regulates Th1 cytokine production, while negatively regulating Th2 cytokine production. Specifically, acute siRNA-mediated knockdown of Dlg1in differentiated Th1 and Th2 cells inhibited IFNγ and TNFα production in Th1 cells, while enhancing IL-4 production in Th2 cells (Fig. 8). While similar results were observed in the conditional knockout system, we did not observe differences in cytokine production in T cells derived from *dlg1* germline knockout mice.

More recently, two groups have shown experimental data which support a model in which Dlg1 couples p38 to NFAT activation and T cell function in primary human T cells to promote distinct signaling pathways in distinct T cell subsets [28], [30]. Work by Zanin-Zhorov et al. has demonstrated that in primary human CD4+CD25+ FoxP3+ T regulatory (Treg) cells, Dlg1 accumulation at the IS is significantly higher than in effector CD4+CD25− T cells. In addition, diminution of Dlg1 expression impaired Treg cell suppression activity, caused a reduction in the amount of Foxp3 per cell, and led to diminished alternative p38 phosphorylation and NFATc1 activation, while enhancing Akt phosphorylation in response to TCR stimulation. These data support our working hypothesis by demonstrating that in human Treg cells, Dlg1 functions as both a positive and negative regulator of discrete signal transduction pathways; promoting alternative p38 activation, while inhibiting Akt activation. Similarly, opposing effects of Dlg1 activity have been observed between TCR-induced p38 and ERK activation [26]. Zanin-Zhorov et al. also elucidate a relationship between Dlg1, NFAT, and Foxp3, which may be of significance since both Foxp3 and NFAT are critical for Treg cell function, commitment, and maintenance [43], [44]. In this study our data, although subtle, hints at a possible relationship between Foxp3 and Dlg1 in Treg cell commitment or maintenance; we observed a trend towards a decreased percentage of CD4+Foxp3+ cells in *dlg1^ko;BG^* germline deficient mice (Fig. 2B). It is interesting to speculate that while compensatory mechanisms may have allowed for T cell development in the mouse models investigated here, Dlg1 may be indispensable for the maintenance as well as function of Treg cell populations. Zanin-Zhorov et al. indicated that Dlg1 recruitment to the IS was diminished in patients with rheumatoid arthritis, suggesting that Dlg1 function and the regulation of the alternative p38 pathway may contribute to dysregulated Treg cell function in rheumatoid arthritis or human autoimmune conditions [28].

While addressing the entire list of discrepancies observed among all the groups investigating the role of Dlg1 in T lymphocytes is beyond the scope of this paper, it seems clear from our and others’ results reported to date that Dlg1 can facilitate or attenuate discrete TCR signals and that its role in regulating T cell functionality can vary within T cell subsets or at particular stages of T cell development [23], [24], [26], [28], [30], [36].

If there are compensatory mechanisms in play, then many of our studies of the role of Dlg1 in T cell function, including surface receptor regulation during primary activation, uropod formation and localization of DPC and IS constituents, and proliferation should be revisited in experimental systems where Dlg1 is acutely knocked out rather than stably deleted during T cell development. Because these data preclude further analysis of Dlg1 using long-term genetic approaches, future efforts to characterize the role of *dlg1* in T cell development and function should make use of genetic systems that allow one to acutely delete *dlg1*. For example, an estrogen-receptor Cre-recombinase (ER-Cre) system where “floxed” alleles can be induced to recombine following exposure to tamoxifen permits targeted and controlled acute Dlg1 ablation might work well. Until the ideal model systems are developed and validated, however, acute knock-down of Dlg1 remains a potent strategy by which to continue exploring the functional significance of Dlg1.

## Materials and Methods

### Ethics Statement

#### Bay Genomics (BG)*- dlg1* knockout mice

All mice were bred and housed at the University of California Division of Laboratory Animal Medicine facility and all experiments were performed in strict accordance with a protocol reviewed and approved by the University of California Animal Research Committee (ARC approval #1996-155-51).

#### Gene Trap (GT)*- dlg1* knockout mice

All mice were bred and housed at the Peter MacCallum Cancer Centre animal facility and all experiments were performed in accordance with the Animal Experimentation Ethics Committee of the Peter MacCallum Cancer Centre (Approval #E349).

#### CD4^Cre^ conditional *dlg1* knockout mice

All studies involving these animals were carried out according to guidelines put forth by the NIH Guide for the Care and Use of Laboratory Animals, as approved under protocol #2008-10-667 by the Children’s Hospital of Philadelphia Institutional Animal Care and Use Committee.

### Mice

#### Bay Genomics (BG)*- dlg1* knockout mice

Mice with a *dlg1* null allele were generated through the Bay Genomics consortium using the gene trap vector pGToLxf. pGToLxf contains a splice acceptor sequence upstream of a β-Geo reporter gene, a fusion of β-galactosidase and neomycin phosphotransferase II. Insertion of the β-Geo cassette disrupts translation of the *dlg1* gene via insertional mutation. The BG-RRN196 ES clone generated by gene trap contains an insertional mutation in the fourth intron of *dlg1* resulting in the production of a fusion transcript consisting of exons 1–4, encoding the first 150 amino acids of Dlg1. Chimeric pups with germline transmission were bred with C57BL/6J to generate a BG-*dlg1* founder line. BG-*dlg1* ^+/−^ founder mice on a mixed 129/C57BL/6J background were crossed with C57BL/6J mice (Jackson Labs) 3+ generations before beginning experiments and maintained as BG-*dlg1* heterozygotes by additional crosses to C57BL/6J mice.

#### Gene Trap (GT)*- dlg1* knockout mice

Mice containing the gene trap insertion allele *dlg1* have been described [37]. GT*-dlg1* mice were crossed with C57BL/6-CD45.2 mice (Walter and Eliza Hall Institute) for at least 10 generations before beginning experiments and maintained as GT-*dlg1* heterozygotes by additional crosses to C57BL/6 mice.

#### CD4^Cre^ conditional *dlg1* knockout mice

Mice homozygous for a floxed *dlg1* gene [38] (generously provided by Dr. R. Huganir, Johns Hopkins University), were crossed with CD4*^Cre^* transgenic mice on the C57Bl/6 background (Taconic Farms) to generate mice with deletion of *dlg1* late in T cell development(*dlg1 ^flox/flox^*:CD4*^Cre^* ). Wild-type littermates (*dlg1 ^flox/flox^*) were used as controls.

### Genotyping

#### BG*- dlg1* knockout mice

BG- *dlg1* ^+/+^ and BG- *dlg1* ^+/−^ mice were genotyped by the absence or presence, respectively, of the β-Geo reporter gene cassette within genomic DNA using a mixture of primers for β-Geo and Tcrd (a control for the presence of genomic DNA): β-Geo forward 5′- CAA ATg gCg ATT ACC gTT gA-3′; β-Geo reverse 5′- TgC CCA gTC ATA gCC gAA TA - 3′; Tcrd forward 5′-CAA ATg TTg CTT gTC Tgg Tg -3′; Tcrd reverse 5′-gTC AgT CgA gTg CAC AgT TT-3′. PCR conditions for 30 cycles were: 94°C for 60 sec; 60°C for 45 sec; 72°C for 60 sec. All successful PCR reactions result in a Tcrd product of 581 base pairs. The presence of β-Geo in BG-*dlg1* ^+/−^, *dlg1^BG;+/−^* or *dlg1^BG;−/−^* samples results in an additional 200 base pair product.

To evaluate β-gal expression levels in donor fetal liver samples and assign a wild-type, heterozygous, or knockout status to the BG-*dlg1* donor pup, 1×10^6^ fetal liver cells were stained using a FluoReporter lacZ Flow Cytometry kit (F1930; Invitrogen/Molecular Probes), according to the manufacturer’s directions. The corresponding heads and bodies of the fetal pups were collected to generate DNA (genomic and cDNA) for further examination by PCR and to extract protein to assess Dlg1 expression by Western blotting, respectively.

“Junctional” primer sets, which span the junction between *dlg1* and the β-Geo insert in cDNA, were also used to differentiate between BG-*dlg1* ^+/+^, BG-*dlg1* ^+/−^, or BG-*dlg1* ^−/−^, donor pups and to confirm genotypes in the lymphoid organs of recipients following adoptive transfer: *dlg1* Forward 5′- gAg Cgg gTT ATT AAC ATA TTT CAg - 3′; *dlg1* PDZ Reverse 5′- CCg CTC gAg CCC TTC TCC ATC TTC ACC TCC -3′; β-Geo Reverse 5′- ATT CAg gCT gCg CAA ATg TTg gg -3′. PCR conditions for 30 cycles were: 94°C for 60 sec; 60°C for 45 sec; 72°C for 60 sec. BG-*dlg1* ^+/+^ and *dlg1^BG;wt^* yield a single product of 1525 bp, BG-*dlg1* ^−/−^ and *dlg1^BG;ko^* yield a single product of 537 bp, and BG-*dlg1* ^+/−^ and *dlg1^BG;+/−^* yield 537 base pairs and 1525 base pair products.

#### GT*- dlg1* knockout mice

For GT- *dlg1*or *dlg1^GT^* mice, the genotype of wild-type, heterozygous or homozygous mutant animals was determined using genomic DNA and primers for the Dlg1 and LacZ reporter gene. Dlg1 forward5′ -GAGTTACCTAAGCCTGGC -3′; Dlg1 reverse 5′- CTGGAATGGGAAACATATAC -3′; LacZ forward 5′-TTGGCGTAAGTGAAGCGAC -3′; LacZ reverse 5′- AGCGGCTGATGTTGAACTG -3′. PCR conditions for 32 cycles were: 94°C 60 sec; 56°C 90 sec; 72°C 90 sec.

### Adoptive Transfer Experiments

#### BG*- dlg1* donor and *dlg1^BG^* HSC recipient mice

Fetal livers from embryonic day 14.5 pups were harvested from BG-*dlg1^+/−^* females crossed to BG-*dlg1^+/−^* males and assigned donor genotypes. 9–14 week old *rag1^−/−^* recipients (B6.129S7-RAG1*^tm1Mom/J^*; Jackson Labs) were sublethally irradiated (400 rads) one day prior to intravenous injection of 0.5×10^6^ donor fetal liver cells. *rag1^−/−^* recipient mice (*dlg1^BG^* ) were analyzed 8–12 weeks post-transfer at which time the bone marrow, thymus, spleen, and lymph nodes were collected. A total of six adoptive transfers were completed with 35 recipient mice examined experimentally (*dlg1^BG;wt^* = 11, *dlg1^BG;+/−^* = 12, and *dlg1^BG;ko^* = 12).

#### GT*- dlg1* donor and *dlg1^GT^* recipient mice

B6-Ptprca recipients (C57BL/6-CD45.1; Walter and Eliza Hall Institute of Medical Research) 8–12 week old were lethally irradiated (550 rads) twice 3 hours apart and injected with 1×10^6^ *dlg1^GT;wt^* and *dlg1^GT;ko^* fetal liver cells.

### Cell Culture

#### *dlg1^BG^* cells and *dlg1^GT^* cells

All primary cells were cultured in complete RPMI media composed of RPMI 1640 supplemented with 10% FBS, sodium pyruvate, GlutaMAX, β-Mercaptoethanol, penicillin, and streptomycin. Total T cells were generated from murine splenocytes +/− lymph nodes, as indicated and red blood cells were removed from single cell suspensions by hypotonic lysis. 293T cells were cultured in DMEM, 10% FBS, non-essential amino acids, sodium pyruvate, penicillin, streptomycin, and glutamate. T lymphoblasts were produced by stimulating spleen cells in complete RPMI 1640 media with 2 µg/ml concanavalin A (Sigma-Aldrich) and 50 U/ml IL-2 (Chiron Corp., USA) for 3 days, and then with 50 U/ml IL-2 for an additional 2 days.

#### dlg1^flox/flox^:CD4^cre^ and jurkat cells

All tissue culture reagents were from Invitrogen. Murine CD4^+^ lymph node T cells were isolated by negative selection using a mixture of anti-MHC class II (M5/114.15.2) and anti-CD8 (2.43) followed by magnetic bead-conjugated goat anti-rat Ig (Qiagen). Labeled cells were removed using magnetic separation. To generate T-depleted splenocytes, red cells were removed from single cell suspensions by hypotonic lysis. Cells were washed and subjected to complement-mediated lysis using α-Thy1.1 (AT83.A) hybridoma supernatant. Dead cells were removed by centrifugation over Histopaque (Sigma). Cells were maintained using DMEM supplemented with 5% FBS, non-essential amino acids, penicillin, streptomycin, HEPES and β-Mercaptoethanol (Sigma). The human T cell line Jurkat E6.1 was maintained in RPMI supplemented with 5% FBS, 5% newborn calf serum, penicillin, streptomycin and GlutaMAX.

### T Cell Expansion

Cells isolated from the spleen and lymph nodes of individual mice were pooled together to expand CD4^+^ and CD8^+^ T-cell populations. Approximately 10×10^6^ cells were stimulated with 5 ug anti-CD3 and 20 ug anti-CD28 in one well of a 6-well plate for 72 hours. Cells were then harvested and re-plated at a concentration of approximately 15×10^6^ cells per well in a 12-well plate in fresh media containing 200 U/ml IL-2 for an additional 48 hrs. Expanded T cell populations were used to assess intracellular cytokine production, phospho-38 phosphorylation, and actin polymerization.

### Reverse Transcription and Quantitative PCR

Total RNA was isolated from purified murine T cells, brain, or Th1 and Th2 cells using Trizol according to the manufacturer’s instructions in order to synthesize cDNA by RTPCR. Resulting cDNA was used to amplify Dlg transcripts with the following primer sets: Dlg1 Forward 5′-CAg AgC AAC CTC TTT CAg gCT T-3′ and Dlg1 Reverse 5′-Tgg ACA TTC TCA ATC TCT gAC A-3′; Dlg2 Forward,5′ CTA CTg TCT ggC AAC AAT ggC A-3′ and Dlg2 Reverse, 5′-TgC AgT ACT gTg CTg AgA ATg A-3′; Dlg3 Forward, 5′-TCg gAC TCg TgA CAg CTg TCT A-3′ and Dlg3 Reverse, 5′-CTC CAT AAT AAT CgT CAC TTA AC-3′; Dlg4 Forward, 5′-TAC CgC TAC CAA gAT gAA gAC AC-3′ and Dlg4 Reverse, 5′-ACT TCA TTG ACA AAC Agg ATg C-3′; Dlg5 Forward, 5′-Tgg CCA Agg AgC Agg ACC ACT T-3′ and Dlg5 Reverse, 5′-gCC TCT CAT AAT CAg gAT TCA gg-3′. PCR products were resolved on a 1% agarose gel, stained with EtBr and visualized with an UV light. An Alpha Imager was used to capture pictures.

Similarly, total RNA from murine CD4+ T cells following Th skewing conditions and transduction with retrovirus expressing control or Dlg1 miRNA was isolated using Trizol according to the manufacturer’s instructions in order to synthesize cDNA by RTPCR. Dlg1 expression and cytokine production was determined by quantitative PCR using 2 ug of total cDNA with the following primer sets: Dlg1 Forward 5′-AgA TCg CAT CAT ATC ggT gAA-3′and Reverse 5′- TCA AAA CgA CTg TAC TCT TCg g-3; IFNγ Forward 5′- gTC AAC AAC CCA CAg gTC CAg -3′and IFNγ Reverse 5′- CCT TTT CCg CTT CCT gAg g -3′; IL4 Forward 5′-ACA ggA gAA ggg ACg CCAT-3′ and IL4 Reverse 5′-gAA gCC CTA CAg ACg AgC T-3′; TNFα Forward 5′- AAT ggC CTC CCT CTC ATC AgT -3′ and TNFα Reverse 5′- gCT ACA ggC TTg TCA CTC gAA TT -3′. A BioRad MyiQ qPCR machine and BioRad iQ5 software was used to generate and quantify data. All samples were normalized to L32 levels (L32 Forward 5′- AAg Cga AAC Tgg Cgg AAA C- 3′and L32 Reverse 5′ –TAA CCg ATg TTg ggC ATC Ag- 3′ in order to determine relative expression levels.

### Cellular Analysis of Cell Populations by Flow Cytometry

Single cell suspensions of 1.5×10^6^ cells were generated from the spleen, lymph nodes, or thymus and surface stained for 20 minutes at 4°C with combinations of following fluorescently-conjugated antibodies at a concentration of 1 ug/ul: CD3, CD4, CD8, CD25, CD44, CD62L, CD69, CD62L, CD44, B220, IgM, and IgD. Intracellular staining for FoxP3 was performed using a FITC anti-mouse FoxP3 staining set according to manufacturer’s directions (eBioscience). Samples were acquired using LSRII or FACSCalibur (BD Biosciences) and analyzed with FlowJo (Treestar) or CellQuest software (BD Biosciences) Dead cells were excluded based on forward versus side scatter analysis.

The hematopoietic composition in peripheral blood was determined by running aliquots of freshly obtained blood samples harvested from the orbital plexus of live mice 8 weeks post-transplantation using sodium heparin-coated capillary tubes (Vitrex Medical AS) on a BAYER ADVIA 120 hematology analyzer (GMI Inc.).

### Cellular Analysis of T Cell Activation

To assay for the modulation of early activation markers on the cell surface, naïve splenocytes (5×10^5^) were stimulated with varying concentrations of plate-bound anti-CD3 and anti-CD28, cultured for 24 hours and labeled with anti-CD8, anti-CD4, anti-CD45.2, anti-CD44, anti-CD69, anti-CD25 and anti-CD62L antibodies for analysis by flow cytometry (FACSDiva) using FlowJo software. Cells were gated for CD4^+^ or CD8^+^. Alternatively, 2×10^5^ enriched CD4^+^ T cells were stimulated with 1 ug/mL each plate-bound anti-CD3 and anti-CD28 for 3 days. Cells were cultured for an additional 2 days and restimulated with plate-bound anti-CD3 and anti-CD28 for 24 hours. Cells were harvested at 24 hours, washed and labeled with anti-CD69, anti-CD25, and anti-CD3 antibodies for analysis by flow cytometry. Dead cells were excluded from analysis based upon forward versus side scatter, and cells were gated in CD4^+^ events.

### Biochemical Analysis of T Cell Activation

#### General tyrosine phosphorylation

For TCR crosslinking, purified *dlg1^flox/flox^* or *dlg1^flox/flox^:CD4^cre^* CD4^+^ T cells were resuspended in RPMI containing 1% FBS, rested on ice in the presence of 10 µg/ml biotinylated anti-CD3 antibody (Biolegend). Stimulation was initiated by the addition of strepavidin (20 µg/ml). Cells were incubated at 37°C, removed at indicated times, and lysed using TTX lysis Buffer (1% Triton X-100, 50 mM TrisHCl, pH 8.0, 50 mM NaCl, 5 mM EDTA, 50 mM NaF, protease inhibitors, 1 mM NaVO_4_). Insoluble material was pelleted at 13,000 rpm for 20 minutes. Protein concentrations were determined using a BCA assay (Pierce). Cell lysates were boiled in sample buffer and separation was performed using SDS-PAGE electrophoresis on Tris-glycine gels with 10% acrylamide. Proteins were transferred to nitrocellulose and blocked in 3% BSA in PBS. Blots were probed with anti-phosphotyrosine (4G10) in 3% BSA in TBST. Blots were probed with AlexaFluor680 donkey anti-mouse Ig (Invitrogen), and visualized using the Odyssey Imager (Licor).

#### Alternative p38 phosphorylation

3×10^6^ expanded T cells from *dlg1^wt;BG^* or *dlg^ko;BG^* mice were rested for 4 hrs at 37°C, and subsequently pretreated with either 10 uM of “InSolution” p38 inhibitor (506148; Calbiochem), 10 uM of ERK inhibitor U0126 (662005; Calbiochem), or DMSO (control) in the presence of complete RPMI media for 30′ minutes at 37°C. Cells were then restimulated with platebound antibody (5 ug anti-CD3 and 20 ug anti-CD28) for 30 minutes at 37°C followed by lysis in TNE buffer (50 mM Tris, 1% Nonidet P-40, 2 mM EDTA, pH 8.0) plus protease (leupeptin and aprotinin 10 ug/ml each, 1 mM PMSF) and phosphatase (1 mM NaVO_4_) inhibitors for 20 minutes on ice. Cellular debris was spun down at 12,000 RPM for 20 minutes at 4°C and cleared lysates were used to assess protein phosphorylation by separation on a 10% SDS-PAGE. Immunoblots were performed with anti-phospho-p38 (Thr180/Tyr182) (3D7) rabbit monoclonal antibody (9215;Cell Signaling), after which the blots were stripped and reprobed with anti-p38α α (C20) rabbit polyclonal antibody (sc-535;Santa Cruz Biotechnologies) to assess loading. Secondary antibodies to detect primary antibodies were donkey anti-rabbit HRP from (sc-2305; Santa Cruz Biotechnology). The induction of p38 phosphorylation was determined by performing densitometry (ImageJ) on immunblots and calculating the relative fold change between an individual unstimulated and stimulated sample.

### RNA-Interference

#### shRNA-Dlg1

For studies in Jurkat T cells, knockdown oligos were synthesized and cloned as a short hairpin into pCMS3.eGFP.H1p. The following targeting sequences were used: Dlg1-Miceli: 5′-TAC ggg AgC AgA TgA TgA A-3′ (adapted from [2]); Dlg1-Harvard: 5′-CCC AAA TCC ATg gAA AAT A-3′ (adapted from [45]). shRNA-containing plasmids (40 µg) were transfected into Jurkat cells growing at log phase using a square wave electroporator (BioRad) as described [46]. Cells were cultured in antibiotic-free media and assayed at 72 hours after transfection. Analysis of GFP^+^ cells by flow cytometry showed >95% transfection efficiency (data not shown). To directly assess protein knockdown, cell lysates were prepared using TTX lysis Buffer as described under methods for “Biochemical analysis of T cell activation”, and separated SDS-PAGE electrophoresis on Tris-glycine gels with 10% acrylamide. Immunoblots were probed with primary antibodies specific for Dlg1 (Santa Cruz) or GAPDH in 3% BSA in TBST, followed by secondary IR800 goat anti-rabbit Ig, (Rockland) or AlexaFluor680 donkey anti-mouse Ig (Invitrogen), and visualized using the Odyssey Imager (Licor).

#### miR-Dlg1

For T helper cell studies, knockdown oligos were synthesized and cloned into the MSCV-based retroviral vector, MGP, where GFP is located downstream of the 5′-LTR. Downstream of the GFP stop codon, the mouse miR-155 expression cassette containing the miR-155 loop sequence and flanking regions was inserted. Anti-sense sequences targeting Dlg1 were designed using the Invitrogen Block-iT polII miR RNAi strategy and cloned into the miR-155 expression cassette, to generate a miR-Dlg1 retroviral construct. The following target sequence was used for MGP-Dlg1∶5′- AgC TTA gAg ACA CCA ACT TAT -3′. A scramble sequence that does not target any known mouse transcript was also cloned into the MGP construct generating a miR-control: 5′-gCg CAg TAC ATT T-3′. To generate retrovirus 293T cells were transfected with pCL-Eco and either miR-control or miR-Dlg1 constructs. Transfection was performed with TransIT 293 (Mirus) as per manufacturer’s instructions. After 48 and 72 hours, viral supernatant was harvested, filtered through a 0.45 um syringe filter and used to spin-infect T helper cells for 90 minutes, at 1250 RPM at 20°C in the presence of 8 ug/ml polybrene (Millipore). After each transduction, retrovirus was removed and cells were cultured in complete RPMI supplemented with 40 U/ml IL-2 for 24 hrs. Analysis of GFP^+^ cells by flow cytometry showed >70% transfection efficiency (data not shown).

### Actin Polymerization

#### Immunofluorescence microscopy –based assay

Analysis of actin polarization was carried out essentially as previously described [47]. Briefly, for Figure 5A, *dlg1^flox/flox^* or *dlg1^flox/flox^:CD4^cre^* CD4^+^ T cells were allowed to conjugate with anti-TCR antibody coated sulfate latex beads in a 2∶1 ratio (beads:cells) for 20 minutes at 37°C, plated on poly-L-lysine coated coverslips, fixed with 3% paraformaldehyde in PBS, permeabilized with 0.3% TX-100, and stained for actin with rhodamine-phalloidin. Cells were then scored for actin localization to the T cell/bead interface by an individual blinded to experimental conditions. For Figure 5D, Jurkat T cells were transfected with either pCMS3.eGFP.H1p empty vector or pCMS3.eGFP.H1p containing a shDlg1 target sequence, and stimulated with SEE-treated or untreated Raji B cells that had been labeled blue with CMAC cell tracker blue. Conjugates were then allowed to settle on poly-L-lysine coated coverslips, fixed, and stained with rhodamine phalloidin as described above. Cell conjugates were then scored for actin localization to the T cell/APC interface by an individual blinded to experimental conditions. Images were collected using a Coolsnap FX-HQ camera (Roper Scientific), and deconvolution and 3-D rendering was performed using Slidebook v4.0 software (3I). 3D images of maximum intensity projections of Z sections spanning the entire cells were compressed and processed using Photoshop CS software (Adobe Systems Inc., USA) to adjust greyscale levels.

#### FACS-based assay

2×10^6^ expanded T cells from *dlg1^wt;BG^* or *dlg1^ko;BG^* mice were stimulated with 5 ug anti-CD3 and 20 ug anti-CD28 of plate-bound antibody in one well of a 6–well dish for 5 or 15 minutes. Cells were harvested and fixed and permeabilized in 200 ul Cytofix/Cytoperm (51–2090KZ; BD Biosciences) over-night at 4°C. Cells were washed in FACS buffer (1× PBS, 3% FCS, and 0.1% sodium azide) and stained for 1 hour at room-temperature with a cocktail of 5 ug/ml FITC-conjugated phalloidin (p-5282; Sigma-Aldrich), anti-CD8b PE and anti-CD4 APC. Cells were then washed twice with FACS buffer and analyzed by flow cytometry (FACSCalibur) using CellQuest software.

### T Cell Polarization Assays

For immunofluorescent imaging, T lymphoblasts were allowed to adhere overnight to Lab-Tec® II chamber slides (Nunc, USA). Cells were washed, fixed in 3.7% paraformaldehyde in PBS, processed with anti-CD43 or anti-CD44_(BD Pharmingen) antibodies to label T cell uropod markers and scored for polarization.

Alternatively, CD4^+^ T cells from *dlg1^flox/flox^* or *dlg1^flox/flox^:CD4^cre^* mice were incubated with anti-TCR antibody-coated beads for 20 minutes, fixed and stained with one of the following antibodies: Ezrin (Cell Signaling), Moesin (Q480; Cell Signaling), CD43 (BD Pharmingen), or PKCζ (Santa Cruz). T cell/bead conjugates were then scored for localization to the DPC or IS interface as above. Quantitation was performed by randomly selecting conjugates containing a T cell contacting a latex bead or blue-dyed B cell. Localization to the IS was defined by the presence of a distinct band at the cell-cell contact site. Localization to the DPC was defined as exclusion of the protein of interest from the cell-cell contact or as capping at the T cell pole opposite the site of TCR engagement. Wherever possible, analysis was performed by an individual blinded to experimental conditions. At least 50 conjugates were scored in each of three experiments. Data represent average +/− standard deviation.

### T Cell Migration Assays

#### Random migration analysis

To assess random migration using time-lapse microscopy, T lymphoblasts cultured overnight in Ibidi chamber slides (Integrated BioDiagnostics, Germany) were followed over 3 hours on a Leica TCS SP5 (Leica Microsystems GmBH, Germany) live-cell microscope equipped with a motorized stage and 37°C heated chamber supplied with CO_2._ Differential interference contrast (DIC) images were captured at 1 min intervals using a Nikon 40X 0.85 N.A. objective. Movement, presented as distance (distances between successive points of each cell from frame to frame), was quantified by individual cell tracking using MetaMorph® 6.3 software.

#### Chemokine-mediated migration assay

*dlg1^flox/flox^* or *dlg1^flox/flox^:CD4^cre^* derived T cell blasts or Jurkat T cells were collected were collected, washed, and resuspended at 20×10^6^ cells/mL in RPMI containing 1% FBS, 25 mM HEPES, 5 mM glutamax and penicillin/streptomycin. Media with or without CCL19 (250 nM) or CXCL12 (10 nM) was plated in bottom wells of 96 well, 3 or 5 um pore ChemoTx® disposable chemotaxis system (NeuroProbe) for T cell blasts or Jurkat T cells, respectively. For some experiments, one side of the chemotaxis chamber membrane was coated with either 20 ug/mL fibronectin or 6 ug/mL rmICAM-1 for two hours, washed, dried, and repeated on the other side. 25 uL of cells were plated on top of the membrane, and plates were incubated at 37°C for 2 hours. Migrated cells were counted by hemocytometer and percent migrated was calculated by dividing by the input cell number. Data represent the average of at least three duplicates +/− standard deviation; statistical significance was calculated using a paired Student’s *t*-test. Data shown is representative of at least 3 independent experiments. *p≤0.05.

### CFSE Proliferation Assay

Total splenocytes from *dlg1^wt;BG^* or *dlg1^ko;BG^* mice were labeled with a final concentration of 1.5 uM CFSE (Invitrogen) and stimulated in 6-well plates coated with varying concentrations of anti-CD3 and anti-CD28 antibodies for 24, 48, or 72 hours. Cells were stained with anti-CD8 antibody (BD Pharmingen) and analyzed by flow cytometry (FACSCalibur) using CellQuest software (BD Biosciences).

Similarly, naïve splenic T lymphocytes isolated by negative bead selection using pan-T cell isolation kits (Miltenyi Biotec) from *dlg1^wt;GT^* or *dlg1^ko;GT^* mice were labeled with 5 µM final concentration of CFSE (Invitrogen), and stimulated with varying concentrations of anti-CD3 antibody in the presence or absence of anti-CD28 and cultured for 62 hours. Cells were washed and labeled with anti-CD8 and anti-CD4 antibodies and analyzed by flow cytometry (LSRII, BD Biosciences) using FlowJo software.

### Intracellular Cytokine Staining

Expanded T cells from *dlg1^wt;BG^* or *dlg^ko;BG^* mice and retrovirally transduced Th1 and Th2 cells were restimulated with plate-bound anti-mouse CD3ε (2 ug/ml) and anti-mouse CD28 (5 ug/ml), or PMA/Ionomycin for 4 or 6 hours, respectively in the presence of GolgiStop (BD Pharmingen). Cells were harvested and surface stained with anti-CD8 or anti-CD4 antibodies as indicated, fixed and permeabilized with Cytofix/Cytoperm (BD Pharmingen) overnight, and subsequently stained with either anti-TNFα (clone MP6-XT22), anti-IFNγ (clone XMG1.2) or anti-IL-4 (clone 11B11) (all BD Pharmingen). Cells were analyzed on a FACSCaliber (BD).

### ELISA

Cytokines were measured by ELISA per antibody manufacturer recommendations (eBioscience).

### Differentiation of T Helper cells

CD4+ T cells were purified from the spleen and lymph nodes of C57Bl/6 mice using anti-CD4 magnetic microbeads (Miltenyi Biotec 130-049-201) according to the manufacturer instructions. Purified CD4+ T cells were stimulated with plate-bound anti-mouse CD3ε (Clone 145-2C11 from BD) at 2 ug/mL and anti-mouse CD28 (Clone 37.51 from BD) at 5 ug/mL under Th1-differentiating conditions (10 ng/mL IL-12, 10 ug/mL anti-IL-4, 40 U/mL IL-2) or Th2-differentiating conditions (20 ng/mL IL-4, 20 ug/mL anti-IFNγ, 20 U/mL IL-2). All antibodies used for Th1/Th2 polarization were from eBiosciences, including neutralizing rat mAb for murine IL-4 (clone 11B11) and IFNγ (clone R4-6A2). Recombinant IL-2, IL-4, and IL-12 were from R and D systems. One week after primary stimulation, cultures were transduced with miR-Dlg1 or miR-control retroviral supernatants, restimulated, and analyzed for cytokine production as described. Data represent average +/− standard error mean and statistical significance was calculated using a paired Student’s *t*-test.

## Supporting Information

Figure S1 **Characterization of** ***dlg*** **gene expression in wild-type T cells.** (A) cDNA from primary mouse T cells and mouse brain was analyzed by PCR using primer pairs specific for 4 distinct *dlg* genes (*dlg* 1–4).
(TIF)

Figure S2 **Schematic for the deletion of** ***dlg1*** **in the germline deficient mouse models.** (A) The RRN196 Dlg1 knockout mouse (BG-*dlg1* ^−/−^) contains a β-galactosidase insertion cassette at the 3′ end of exon 4 expected to result in a truncated 105 amino acid Dlg1-β-Geo fusion protein. (B) The GT-*dlg1* knockout mouse contains a β-galactosidase insertion cassette between the Dlg1 PDZ3 and SH3 domain expected to result in a truncated 549 amino acid Dlg1-β-Geo fusion protein. (C) *Top*, Diagram of primers sets for junctional PCR to differentiate between BG-*dlg1* ^+/+^, BG-*dlg1* ^+/−^, or BG-*dlg1* ^−/−^ donor pups. Expected PCR products are as follows: BG-*dlg1* ^+/+^ = 1525 bp;BG-*dlg1* ^−/−^ = 525 bp. *Bottom*, Representative agarose gel showing differential products of junctional PCR from BG-*dlg1* ^+/+^, BG-*dlg1* ^+/−^, or BG-*dlg1* ^−/−^ fetal pups (n = 6 independent experiments).
(TIF)

Figure S3 **Hematopoiesis is not altered in** *dlg1^ko;GT^* **mice.** Eight weeks following reconstitution, peripheral blood from *dlg1^wt;GT^* or *dlg1^ko;GT^* mice was collected and analyzed for total blood composition using BAYER ADVIA 120 hematology analyzer. Data are expressed as mean ± SD. (n = 10 mice per genotype).
(TIF)

Table S1 **Thymic and splenic cellularity and composition are not altered in in** *dlg1^ko;GT^* **mice.** Total thymocyte and splenocyte cellularity (1×10^6^) and percentage of cellular subsets was determined by cell counts on a hemocytometer and flow cytometric analysis of indicated cell surface markers. Shown are averages ± standard deviations of 12 *dlg1^wt;GT^* and *dlg1^ko;GT^* mice.
(TIF)
