# Supplementary figures and images for: Characterization of In Vivo Dlg1 Deletion on T Cell Development and Function
Source: PLoS One. 2012 Sep 18;7(9):e45276. doi: 10.1371/journal.pone.0045276 (PMC3445470; doi:10.1371/journal.pone.0045276)

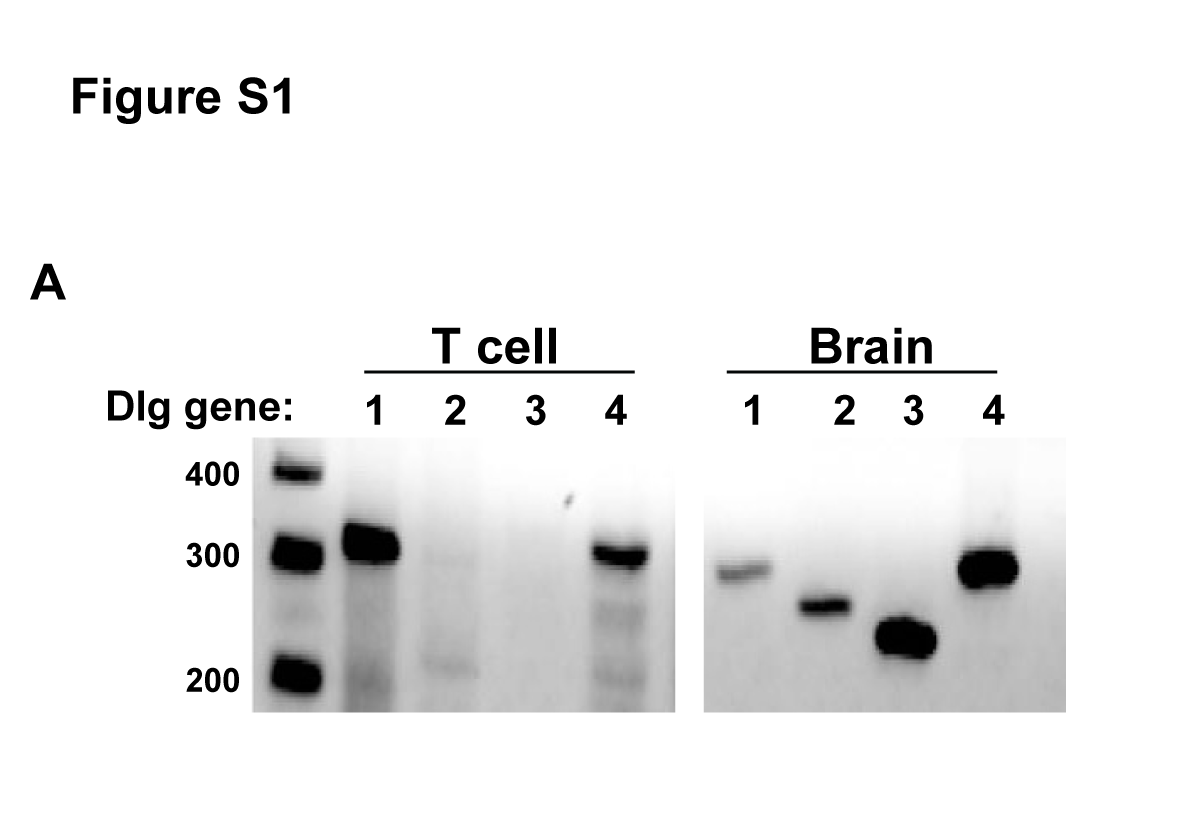

Supplement: Figure S1 — Characterization of dlg gene expression in wild-type T cells. (A) cDNA from primary mouse T cells and mouse brain was analyzed by PCR using primer pairs specific for 4 distinct dlg genes (dlg 1–4). (TIF) [file pone.0045276.s001.tif]

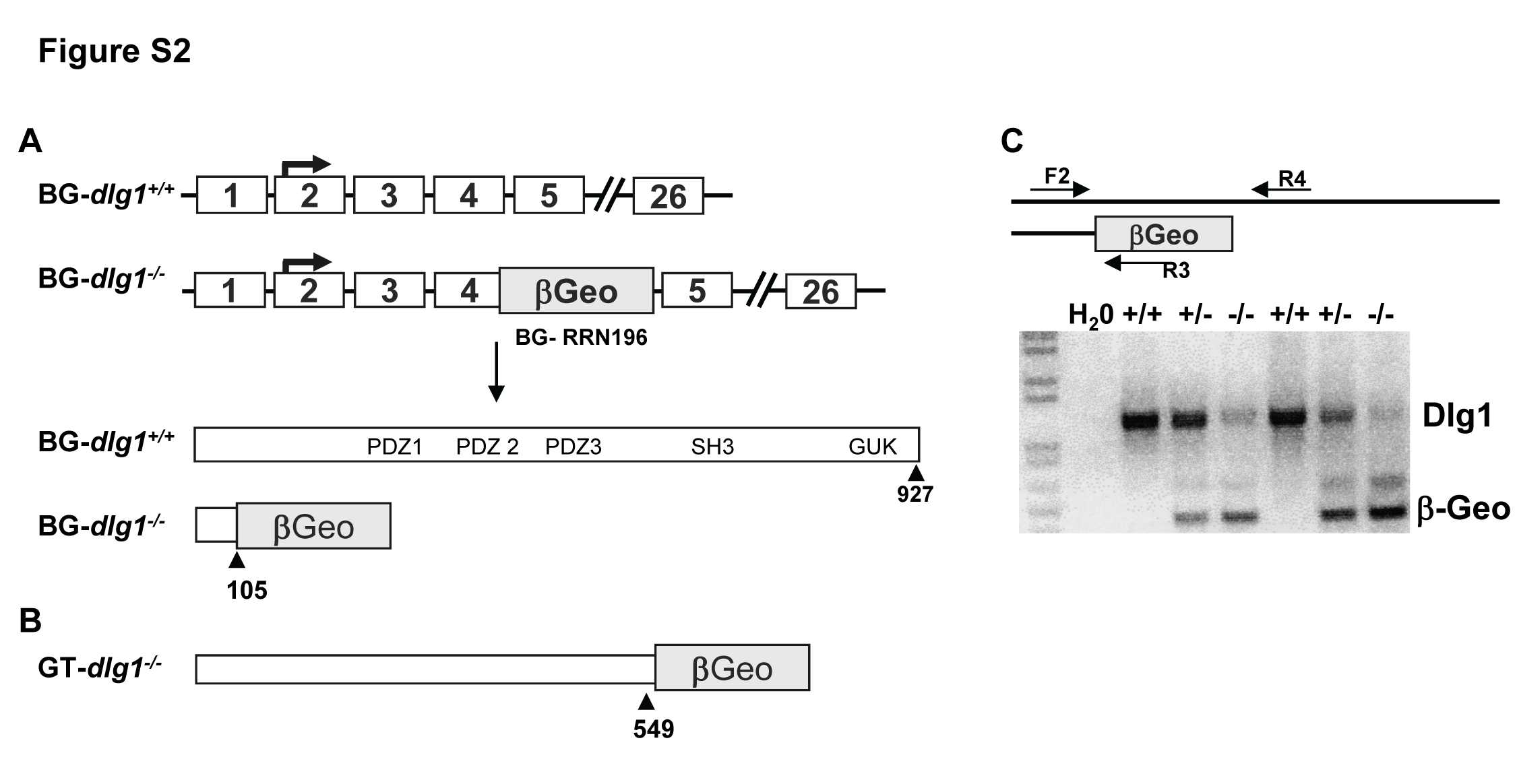

Supplement: Figure S2 — Schematic for the deletion of dlg1 in the germline deficient mouse models. (A) The RRN196 Dlg1 knockout mouse (BG-dlg1 −/−) contains a β-galactosidase insertion cassette at the 3′ end of exon 4 expected to result in a truncated 105 amino acid Dlg1-β-Geo fusion protein. (B) The GT-dlg1 knockout mouse contains a β-galactosidase insertion cassette between the Dlg1 PDZ3 and SH3 domain expected to result in a truncated 549 amino acid Dlg1-β-Geo fusion protein. (C) Top, Diagram of primers sets for junctional PCR to differentiate between BG-dlg1 +/+, BG-dlg1 +/−, or BG-dlg1 −/− donor pups. Expected PCR products are as follows: BG-dlg1 +/+ = 1525 bp;BG-dlg1 −/− = 525 bp. Bottom, Representative agarose gel showing differential products of junctional PCR from BG-dlg1 +/+, BG-dlg1 +/−, or BG-dlg1 −/− fetal pups (n = 6 independent experiments). (TIF) [file pone.0045276.s002.tif]

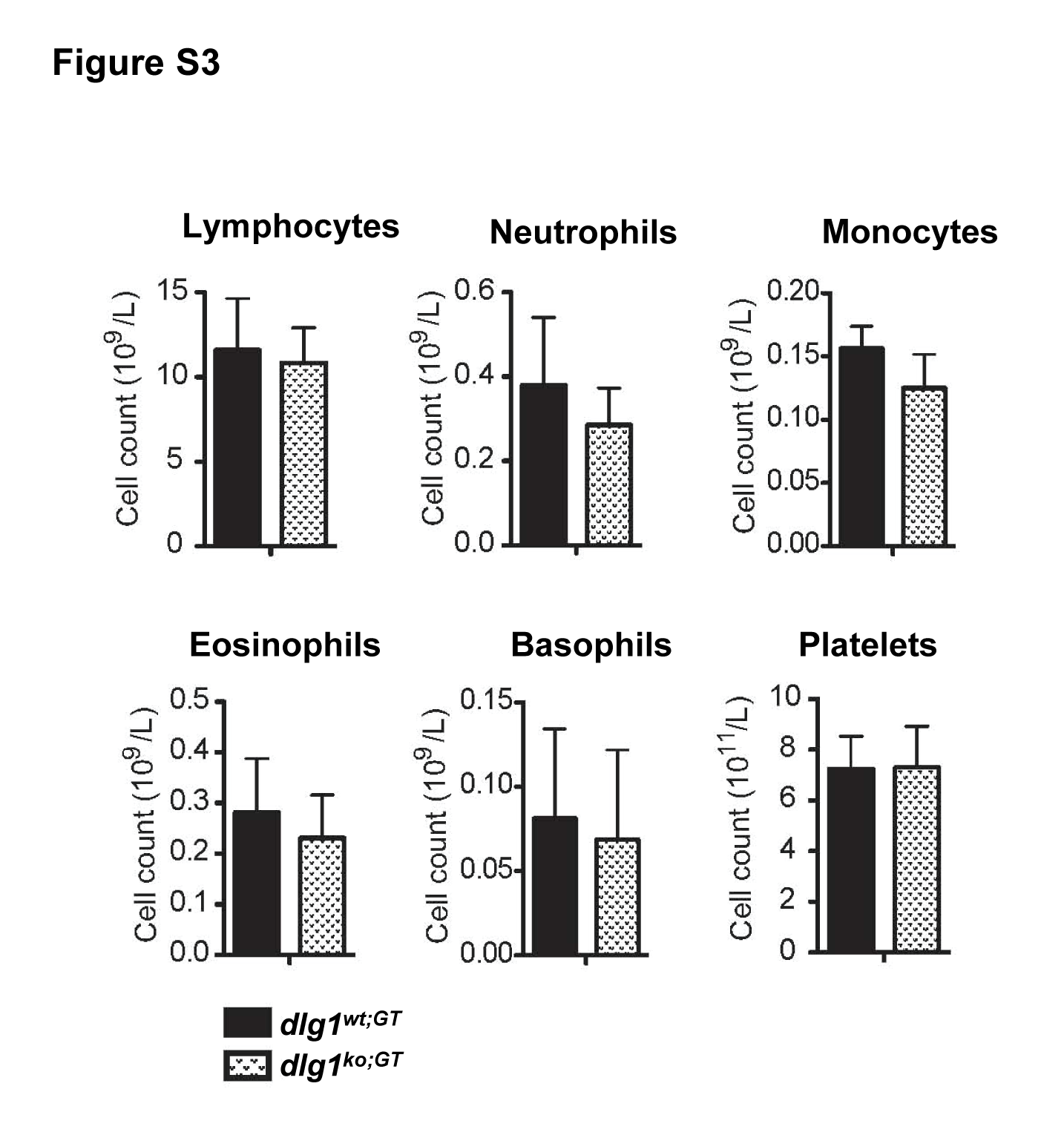

Supplement: Figure S3 — Hematopoiesis is not altered in dlg1ko;GT mice. Eight weeks following reconstitution, peripheral blood from dlg1wt;GT or dlg1ko;GT mice was collected and analyzed for total blood composition using BAYER ADVIA 120 hematology analyzer. Data are expressed as mean ± SD. (n = 10 mice per genotype). (TIF) [file pone.0045276.s003.tif]

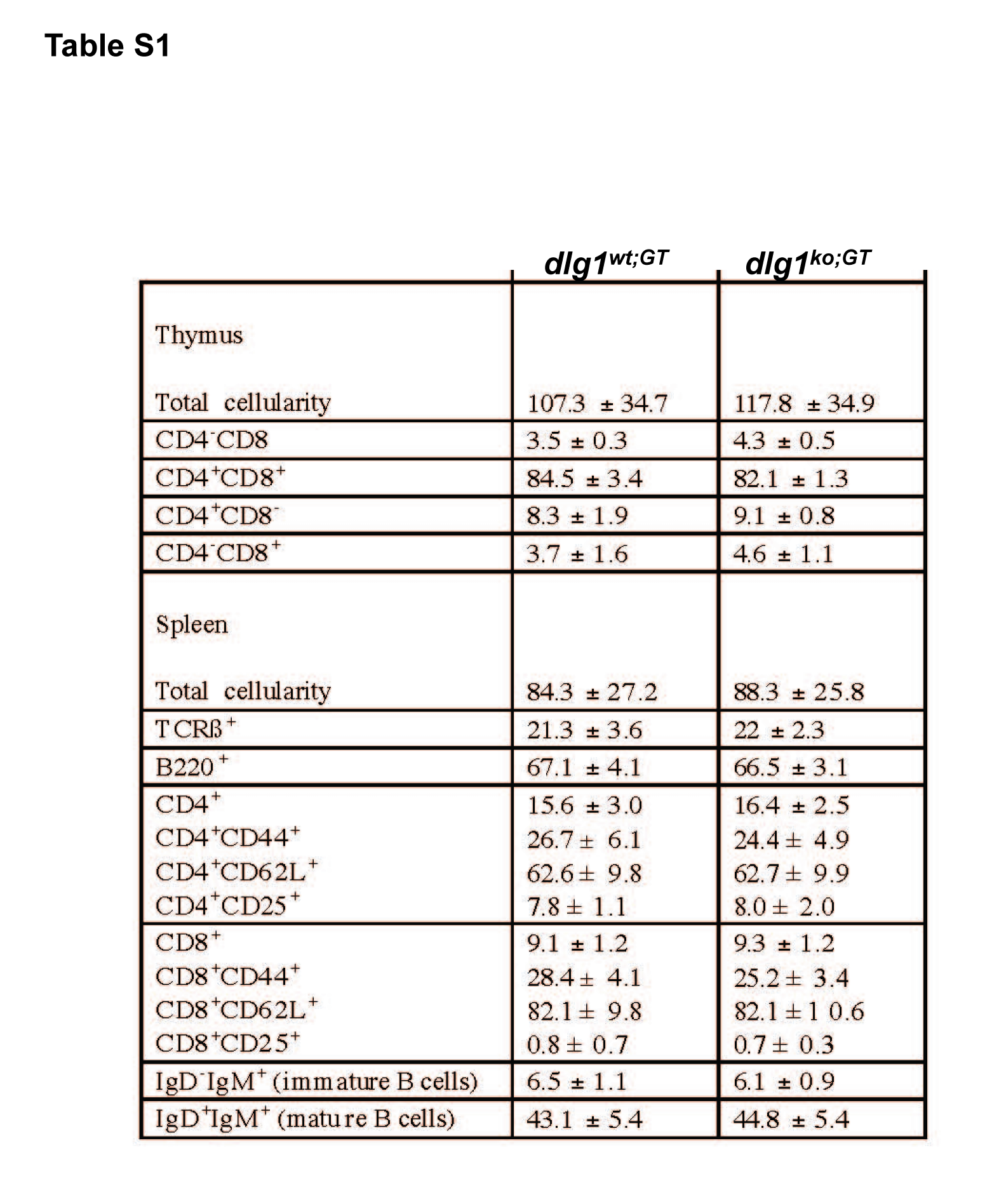

Supplement: Table S1 — Thymic and splenic cellularity and composition are not altered in in dlg1ko;GT mice. Total thymocyte and splenocyte cellularity (1×10^6^) and percentage of cellular subsets was determined by cell counts on a hemocytometer and flow cytometric analysis of indicated cell surface markers. Shown are averages ± standard deviations of 12 dlg1wt;GT and dlg1ko;GT mice. (TIF) [file pone.0045276.s004.tif]
